# Distinct single‐nucleus RNA‐seq changes among non‐neuronal cells in ADNC, LATE‐NC, and mixed pathologies

**DOI:** 10.1002/alz.71810

**Published:** 2026-09-23

**Authors:** Qi Qiao, Yuriko Katsumata, Hsin‐Yu Lai, Kevin Z. Lin, Steven A. Claas, Mariano I. Gabitto, Josh M. Morganti, Peter T. Nelson, Shubhabrata Mukherjee, David W. Fardo

**Affiliations:** ^1^ Department of Biostatistics College of Public Health University of Kentucky Lexington Kentucky USA; ^2^ Sanders‐Brown Center on Aging and Alzheimer's Disease Research Center University of Kentucky Lexington Kentucky USA; ^3^ Division of Biomedical Informatics University of Kentucky College of Medicine Lexington Kentucky USA; ^4^ Allen Institute for Brain Science Seattle Washington USA; ^5^ Department of Pathology and Laboratory Medicine University of Kentucky College of Medicine Lexington Kentucky USA; ^6^ Department of Biostatistics University of Washington Seattle Washington USA; ^7^ Department of Statistics University of Washington Seattle Washington USA; ^8^ Department of Neuroscience University of Kentucky Lexington Kentucky USA; ^9^ Department of Medicine University of Washington Seattle Washington USA

**Keywords:** aging brain, Alzheimer's disease, comorbid neuropathology, differential gene expression, glial cell types, limbic‐predominant age‐related transactive response DNA binding protein 43 kDa encephalopathy, single‐nucleus RNA sequencing, transcriptomics

## Abstract

**INTRODUCTION:**

Limbic‐predominant age‐related transactive response DNA binding protein 43 kDa encephalopathy neuropathological change (LATE‐NC) frequently co‐occurs with Alzheimer's disease neuropathologic change (ADNC), complicating classification. Cell type–specific molecular features distinguishing LATE‐NC‐dominant, ADNC‐dominant, and mixed LATE‐NC and ADNC pathology remain incompletely characterized.

**METHODS:**

We analyzed single‐nucleus RNA sequencing data from the Seattle Alzheimer's Disease Brain Cell Atlas consortium, focusing on non‐neuronal cells in the middle temporal gyrus (MTG). Donors were stratified into LATE‐NC dominant, ADNC dominant, and mixed LATE‐NC and ADNC. Differential expression and gene set enrichment analyses were performed across glial and vascular cell supertypes.

**RESULTS:**

Distinct transcriptional patterns differentiated pathologies. Astrocytes, microglia, and oligodendrocytes exhibited differential expression between LATE‐NC‐ and ADNC‐dominant cases. Representative genes and pathways highlighted these distinctions, with LATE‐NC dominant showing enrichment in signaling and RNA regulation, ADNC dominant in mitochondrial dysfunction, and mixed pathology in translational dysregulation and lipid remodeling, suggesting a distinct biological state.

**DISCUSSION:**

Findings highlight biological heterogeneity and non‐neuronal molecular features relevant to disease classification and future research.

**CLINICAL TRIAL REGISTRATION INFORMATION:**

Not applicable. This study is a secondary analysis of publicly available Seattle Alzheimer's Disease Brain Cell Atlas (SEA‐AD) data.

## Highlights

- Cell type–specific transcriptional patterns distinguish limbic‐predominant age‐related transactive response DNA binding protein 43 kDa encephalopathy neuropathological change (LATE‐NC), Alzheimer's disease neuropathologic change (ADNC), and LATE‐NC and ADNC.
- LATE‐NC is associated with cellular signaling, lipid metabolic, and RNA regulatory pathways.
- ADNC is dominated by mitochondrial dysfunction and proteostasis‐related stress responses.
- Mixed LATE‐NC and ADNC pathology exhibits distinct translational dysregulation and convergent cellular stress responses.
- Disease‐associated genes show group‐specific expression across glial cell types.

## 1 BACKGROUND

Limbic‐predominant age‐related transactive response DNA binding protein 43 kDa (TDP‐43) encephalopathy (LATE) has emerged as a highly prevalent neurodegenerative condition that affects many older adults and shares substantial clinical overlap with Alzheimer's disease (AD).^1^, ^2^, ^3^, ^4^, ^5^ LATE neuropathological change (LATE‐NC) is defined by the accumulation of phosphorylated TDP‐43 pathology across limbic brain regions, which results in amnestic symptoms that are difficult to distinguish from those of AD in clinical settings.^1^, ^6^, ^7^, ^8^, ^9^ Moreover, LATE‐NC is a prevalent condition and frequently co‐occurs with AD neuropathologic change (ADNC).^1^, ^4^ Among the ≈ 20% of individuals who lack ADNC in advanced age, ≈ 25% have LATE‐NC, whereas in persons with severe ADNC, ≈ 50% have LATE‐NC.^10^ Together, these observations indicate a complex pathological landscape that is not captured by routine clinical diagnoses.^4^

The conflation of LATE‐NC and ADNC with “probable AD” is common in both research and clinical settings because of the shared clinical features.^11^, ^12^ This lack of diagnostic specificity has significant implications.^13^, ^14^, ^15^ For example, co‐occurring LATE‐NC and ADNC may represent a biologically distinct entity with unique molecular underpinnings, suggesting that therapies optimized for amyloid beta (Aβ) or tau may not sufficiently address the cellular mechanisms underlying TDP‐43 pathology.^4^, ^15^ A deeper understanding of the underlying biological heterogeneity among LATE‐NC–dominant, ADNC–dominant, and comorbid LATE‐NC and ADNC cases may help improve differential diagnosis and inform future biomarker discovery and targeted therapeutic strategies.

Although large‐scale genetic and neuropathologic studies have identified risk variants and implicated numerous candidate genes for ADNC and LATE‐NC,^16^, ^17^, ^18^, ^19^, ^20^ additional information is needed to understand the cell type–specific processes that drive selective vulnerability, disease progression, and gene‐regulatory disruption. Single‐nucleus RNA sequencing (snRNA‐seq) helps address these gaps by enabling transcriptomic profiling of individual brain cell types and subtypes, allowing researchers to link genetic risk to specific cellular contexts of gene regulation and dysregulation.^21^, ^22^, ^23^, ^24^, ^25^, ^26^, ^27^, ^28^, ^29^ Previous single‐cell studies in ADNC have highlighted widespread alterations in neuronal subtypes, microglia, and astrocytes.^30^, ^31^, ^32^, ^33^, ^34^, ^35^, ^36^, ^37^ However, to our knowledge, no study has systematically compared LATE‐NC dominant (i.e., lacking intermediate or high ADNC), ADNC dominant (i.e., lacking LATE‐NC), and mixed LATE‐NC and ADNC at the single‐cell level within the same brain regions.

We leveraged high‐quality snRNA‐seq data from the Seattle Alzheimer's Disease Brain Cell Atlas (SEA‐AD) consortium,^30^ which provides deeply profiled cellular transcriptomes across the middle temporal gyrus (MTG) and prefrontal cortex (A9). We stratified donors into ADNC–dominant (ADNC), LATE‐NC–dominant (LATE‐NC), and mixed LATE‐NC and ADNC (LATE‐NC&ADNC) groups using neuropathological criteria for ADNC severity and LATE‐NC staging, enabling systematic pairwise comparisons of the transcriptomic landscapes across the three disease categories.

We performed differential expression analysis and gene set enrichment analysis (GSEA) at the cell supertype level^30^ to identify cell type–level molecular signatures that distinguish or are shared among LATE‐NC, ADNC, and LATE‐NC&ADNC cases. By integrating gene‐level and pathway‐level analyses, we examined whether unique or overlapping transcriptional programs across these groups may contribute to diagnostic uncertainty and reflect distinct underlying disease biology. Our findings highlight both shared and pathology‐specific molecular processes across cell types and brain regions, and also provide a framework to inform future efforts in differential diagnosis, disease‐associated gene prioritization, and therapeutic research. This work advances the development of more precise diagnostic frameworks informed by the high prevalence of co‐occurring pathology in the aging brain.

**RESEARCH IN CONTEXT**

1. **Systematic review**: The authors reviewed the literature using traditional (e.g., PubMed) sources. While prior studies have characterized glial alterations in Alzheimer's disease (AD), few single‐cell and neuropathologic investigations have examined cell type–specific changes across AD neuropathologic change (ADNC), limbic‐predominant age‐related transactive response DNA binding protein 43 kDa encephalopathy neuropathological change (LATE‐NC), and their co‐occurring pathologies. Direct single‐cell comparisons among LATE‐NC dominant, ADNC dominant, and mixed pathology remain limited.
2. **Interpretation**: This study identifies distinct, cell type–specific transcriptional patterns across neuropathologic groups. LATE‐NC is associated with signaling and lipid metabolic pathways, ADNC with mitochondrial dysfunction and proteostasis stress, and mixed pathology with a non‐intermediate, convergent stress‐response state, supporting its classification as a distinct biological state.
3. **Future directions**: The discovered genes and pathways could contribute to further study aiming at the differentiation of LATE‐NC, ADNC, and mixed pathology in research and clinical settings.

## 2 METHODS

### 2.1 Donor stratification by ADNC and LATE‐NC stage and donor characteristics

**FIGURE 1:**
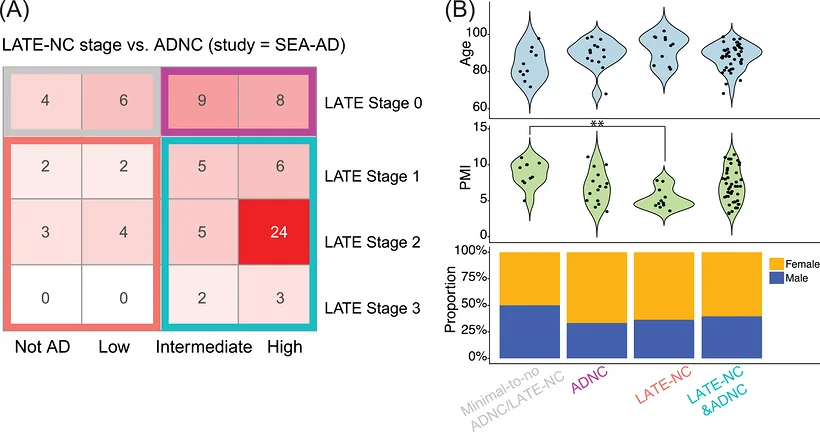
Cohort characteristics, including (A) pathological classification distribution of research participants designated as minimal‐to‐no ADNC/LATE‐NC (gray border), ADNC‐dominant (purple border), LATE‐NC‐dominant (salmon border), and mixed LATE‐NC&ADNC (teal border). B, Distribution of age at death, PMI, and sex proportion of the four neuropathologically defined groups. Age at death and PMI were compared using Kruskal–Wallis tests; sex distribution was compared using the Fisher exact test. PMI differed significantly across groups, driven by the minimal‐to‐no ADNC/LATE‐NC versus LATE‐NC comparison after Benjamini–Hochberg correction. After excluding the minimal‐to‐no ADNC/LATE‐NC group, which was not included in downstream analyses, PMI was no longer significantly different among the remaining MTG groups. AD, Alzheimer's disease; ADNC, Alzheimer's disease neuropathologic change; LATE‐NC, limbic‐predominant age‐related transactive response DNA binding protein 43 kDa encephalopathy neuropathological change; MTG, middle temporal gyrus; PMI, *post mortem* interval; SEA‐AD, Seattle Alzheimer's Disease Brain Cell Atlas.

Donor‐level neuropathological and clinical data were obtained from SEA‐AD files. Donors were divided into four groups based on ADNC and LATE‐NC stage levels using the “Overall AD neuropathological change”^38^ and “LATE”^1^ metadata fields, respectively. ADNC was defined following National Institute on Aging–Alzheimer's Association (NIA–AA) guidelines, which integrate Thal Aβ plaque phase (A score), Braak neurofibrillary tangle stage (B score), and Consortium to Establish a Registry for Alzheimer's Disease (CERAD) neuritic plaque density (C score).^38^ The LATE‐NC stage was defined according to consensus guidelines.^1^ ADNC status was dichotomized into not AD/Low versus Intermediate/High, and LATE‐NC was None versus Stages 1 to 3. Donors annotated as “Unclassifiable” for LATE‐NC were excluded from downstream analyses. This resulted in the following neuropathological subgroups: minimal‐to‐no ADNC/LATE‐NC (*n* = 10), LATE‐NC dominant (*n* = 11), ADNC dominant (*n* = 17), and mixed LATE‐NC&ADNC (*n* = 45; Figure 1A). Table 1 summarizes demographic, clinical, and neuropathological variables across the four groups; quantitative neuropathology marker comparisons are in Tables S1 and S2 and Figure S1 in supporting information.

**TABLE 1 alz71810-tbl-0001:** Demographic and neuropathologic characteristics of MTG samples across the ADNC‐dominant, LATE‐NC‐dominant, LATE‐NC&ADNC groups, and the combined sample.

Characteristics	Minimal‐to‐no ADNC/LATE‐NC (*n* = 10)	ADNC‐dominant (*n* = 17)	LATE‐NC‐dominant (*n* = 11)	LATE‐NC&ADNC (*n* = 45)	Overall (*n* = 83)
**Sex**					
Female	5 (50.0%)	12 (70.6%)	7 (63.6%)	27 (60.0%)	51 (61.4%)
Male	5 (50.0%)	5 (29.4%)	4 (36.4%)	18 (40.0%)	32 (38.6%)
**Age at death (year)**					
Mean (SD)	84 (8.46)	88.2 (9.39)	92.5 (7.58)	89.5 (6.61)	89 (7.77)
Median (minimum, maximum)	82 (72, 98)	91 (65, 99)	95 (81, 102)	90 (70, 100)	90 (65, 102)
**PMI**					
Mean (SD)	8.8 (1.78)	7 (2.24)	5.3 (1.44)	7 (2.33)	7 (2.29)
Median (minimum, maximum)	9 (5, 11)	6.9 (3.50, 11.1)	4.7 (3.60, 7.83)	6.9 (3.20, 11.42)	6.9 (3.20, 11.42)
**Self‐reported race**					
White	10 (100.0%)	16 (94.1%)	10 (90.9%)	44 (97.8%)	80 (96.4%)
Mixed	0 (0.0%)	1 (5.9%)	1 (9.1%)	1 (2.2%)	3 (3.6%)
***APOE* ε4 alleles**					
0	10 (100.0%)	11 (64.7%)	11 (100.0%)	26 (57.8%)	58 (69.9%)
1	0 (0.0%)	5 (29.4%)	0 (0.0%)	14 (31.1%)	19 (22.9%)
2	0 (0.0%)	1 (5.9%)	0 (0.0%)	5 (11.1%)	6 (7.2%)
**ADNC**					
Not AD	4 (40.0%)	0 (0.0%)	5 (45.5%)	0 (0.0%)	9 (10.8%)
Low	6 (60.0%)	0 (0.0%)	6 (54.5%)	0 (0.0%)	12 (14.5%)
Intermediate	0 (0.0%)	9 (52.9%)	0 (0.0%)	12 (26.7%)	21 (25.3%)
High	0 (0.0%)	8 (47.1%)	0 (0.0%)	33 (73.3%)	41 (49.4%)
**LATE‐NC**					
LATE Stage 0	10 (100.0%)	17 (100.0%)	0 (0.0%)	0 (0.0%)	27 (32.5%)
LATE Stage 1	0 (0.0%)	0 (0.0%)	4 (36.4%)	11 (24.4%)	15 (18.1%)
LATE Stage 2	0 (0.0%)	0 (0.0%)	7 (63.6%)	29 (64.4%)	36 (43.4%)
LATE Stage 3	0 (0.0%)	0 (0.0%)	0 (0.0%)	5 (11.1%)	5 (6.0%)
**Thal**					
Thal 0	4 (40.0%)	0 (0.0%)	5 (45.5%)	0 (0.0%)	9 (10.8%)
Thal 1	4 (40.0%)	0 (0.0%)	1 (9.1%)	0 (0.0%)	5 (6.0%)
Thal 2	1 (10.0%)	0 (0.0%)	4 (36.4%)	2 (4.4%)	7 (8.4%)
Thal 3	1 (10.0%)	4 (23.5%)	0 (0.0%)	7 (15.6%)	12 (14.5%)
Thal 4	0 (0.0%)	9 (52.9%)	1 (9.1%)	19 (42.2%)	29 (34.9%)
Thal 5	0 (0.0%)	4 (23.5%)	0 (0.0%)	17 (37.8%)	21 (25.3%)
**Braak**					
Braak 0	2 (20.0%)	0 (0.0%)	0 (0.0%)	0 (0.0%)	2 (2.4%)
Braak II	3 (30.0%)	0 (0.0%)	1 (9.1%)	0 (0.0%)	4 (4.8%)
Braak III	3 (30.0%)	2 (11.8%)	0 (0.0%)	1 (2.2%)	6 (7.2%)
Braak IV	2 (20.0%)	7 (41.2%)	9 (81.8%)	5 (11.1%)	23 (27.7%)
Braak V	0 (0.0%)	5 (29.4%)	1 (9.1%)	28 (62.2%)	34 (41.0%)
Braak VI	0 (0.0%)	3 (17.6%)	0 (0.0%)	11 (24.4%)	14 (16.9%)
**CERAD score**					
Absent	8 (80.0%)	1 (5.9%)	7 (63.6%)	0 (0.0%)	16 (19.3%)
Sparse	1 (10.0%)	3 (17.6%)	4 (36.4%)	4 (8.9%)	12 (14.5%)
Moderate	0 (0.0%)	5 (29.4%)	0 (0.0%)	21 (46.7%)	26 (31.3%)
Frequent	1 (10.0%)	8 (47.1%)	0 (0.0%)	20 (44.4%)	29 (34.9%)

Abbreviations: ADNC, Alzheimer's disease neuropathologic change; *APOE*, apolipoprotein E; CERAD, Consortium to Establish a Registry for Alzheimer's Disease; LATE‐NC, limbic‐predominant age‐related transactive response DNA binding protein 43 kDa encephalopathy neuropathological change; MTG, middle temporal gyrus; PMI, *post mortem* interval; SD, standard deviation.

Differences in age at death and *post mortem* interval (PMI) across neuropathological groups were assessed using Kruskal–Wallis tests. Sex distribution across groups was assessed using Fisher exact test. When global tests were significant, post hoc pairwise comparisons were performed using Wilcoxon rank‐sum tests for continuous variables and Fisher exact tests for categorical variables, with Benjamini–Hochberg correction for multiple comparisons. Because the minimal‐to‐no ADNC/LATE‐NC group was not included in downstream analyses, PMI was additionally reassessed after excluding this group.

### 2.2 snRNA‐seq analysis

#### 2.2.1 Data source and acquisition

We analyzed processed single‐nucleus transcriptomic data from the SEA‐AD consortium.^30^, ^39^ Human brain tissue was collected at rapid autopsy. We downloaded the processed SEA‐AD single‐cell profiling data from the publicly accessible Registry of Open Data on Amazon Web Service Allen Institute SEA‐AD data portal and did not reprocess raw sequencing reads. The processed single‐cell profiling data are available in the public AWS S3 bucket (s3://sea‐ad‐single‐cell‐profiling/) and can be accessed without an AWS account using –no‐sign‐request option. The Python package SCANPY v1.11.2^40^ and R package Seurat v5.2.0^41^ were used to convert the H5AD files to Seurat objects.

#### 2.2.2 Quality control of processed SEA‐AD nuclei

We used the processed SEA‐AD MTG H5AD files and associated subclass/supertype annotations provided by the SEA‐AD study and did not reprocess any raw sequencing data.^30^ Therefore, the primary nucleus‐level quality control (QC) and annotation filtering followed the SEA‐AD pipeline.^30^ Briefly, SEA‐AD removed transcriptomic nuclei with < 500 detected genes before supertype mapping. Low‐quality nuclei or metacells were further evaluated using group doublet scores, fraction of mitochondrial reads, number of detected genes, and donor entropy (used scipy.stats.entropy with default parameters, version 1.8.1). These thresholds were adjusted within each subclass to account for differences in RNA content across cell types.^30^ In the present study, we retained cells from the processed SEA‐AD object after these QC steps. As a post hoc QC summary of the final analyzed cells, we calculated mitochondrial read percentage in the retained Seurat object. Across 231,373 cells from 30 supertypes, the median mitochondrial percentage was 0.09% (interquartile range: 0.03%–0.24%; mean: 0.24%; maximum: 4.99%), and no cells exceeded 5%, 10%, or 20% mitochondrial reads. The highest supertype‐level median mitochondrial percentage was 1.31% in monocytes, and the highest donor‐level median mitochondrial percentage was 1.43%.

#### 2.2.3 SEA‐AD Uniform Manifold Approximation and Projection embedding and visualization

We used the Uniform Manifold[Table alz71810-tbl-0001] Approximation and Projection (UMAP) coordinates provided in the processed SEA‐AD H5AD file from the SEA‐AD study.^30^ In SEA‐AD, nuclei were hierarchically mapped to class, subclass, and supertype labels using single‐cell variational inference (scVI)/single‐cell annotation using variational inference (scANVI). Briefly, SEA‐AD transcriptomic nuclei with < 500 detected genes were excluded before mapping. The scVI/scANVI models were trained using selected transcriptomic features, including 2000 highly variable genes and 500 differentially expressed genes from the reference data, and the donor identity and number of detected genes were included as covariates. The UMAP embedding was generated from SEA‐AD subclass‐specific scANVI latent spaces by constructing a nearest‐neighbor graph with scanpy.pp.neighbors() using default settings, followed by two‐dimensional UMAP with scanpy.tl.umap() using default settings. We used the provided embedding only for visualization and did not use the UMAP coordinates for differential expression testing.

#### 2.2.4 SEA‐AD subclass and supertype annotation by reference label transfer

Cell subclass and supertype annotations were adopted from the SEA‐AD integrated multimodal cell atlas.^30^, ^32^ In the SEA‐AD study,^30^ nuclei were mapped to a hierarchical reference taxonomy using an iterative scVI/scANVI framework in which labels were predicted sequentially at the class, subclass, and supertype levels. SEA‐AD transcriptomic nuclei with < 500 detected genes were excluded before mapping, and predicted labels were evaluated using scANVI prediction probabilities and marker‐gene signature scores. Only non‐neuronal populations were analyzed in this study, including astrocytes, microglia, oligodendrocytes, oligodendrocyte precursor cells (OPCs), endothelial cells, and vascular leptomeningeal cells (VLMCs). These populations were further annotated into distinct cell supertypes based on the SEA‐AD reference annotations.^30^, ^32^ In the present study, we retained the SEA‐AD–provided subclass and supertype annotations. To evaluate the consistency of these annotations, we visualized canonical marker genes for major non‐neuronal subclasses using dot plots. For supertype‐level annotations, we visualized representative marker genes adopted from the SEA‐AD study using violin plots within each subclass.^30^

#### 2.2.5 Donor‐level cell proportion analysis

Donor‐level non‐neuronal cell‐supertype proportions were analyzed using a beta regression framework following a previously published paper.^42^ For each donor, the proportion of each supertype was calculated as the number of cells assigned to that supertype divided by the total number of retained non‐neuronal cells from the same donor. Proportions were modeled separately for each supertype using beta regression with a logit link implemented in glmmTMB(), with neuropathological group as the main variable of interest and age at death, sex, PMI, and log10‐transformed total donor cell number included as covariates. Proportions equal to 0 or 1 were adjusted using a Smithson and Verkuilen‐style transformation before model fitting to satisfy beta regression assumptions. Global group effects were assessed using type III analysis of deviance, and pairwise group contrasts were estimated using emmeans() with Benjamini–Hochberg correction for multiple testing. The custom R script used for this analysis was deposited in Zenodo (https://doi.org/10.5281/zenodo.20721887).

#### 2.2.6 Assessment of neuronal RNA contamination

To assess potential neuronal RNA contamination in non‐neuronal cell populations,^43^, ^44^, ^45^ we examined the expression of canonical neuronal marker genes across all cell classes. Mean expression levels in non‐neuronal populations were compared to those in neuronal populations to evaluate the typical background expression observed in snRNA‐seq datasets. Detailed diagnostic analyses are provided in Supplementary Methods in supporting information.

#### 2.2.7 Differentially expressed genes analysis

The differentially expressed gene (DEG) analysis was performed on non‐neuronal cell supertypes with at least 50 cells in each neuropathologic group. Counts per cell (CPC) for each gene were calculated as the total unique molecular identifier (UMI) counts divided by the number of cells in the qualified cell supertype as a QC step before the DEG analysis. Genes with CPC < 0.1% were excluded from further analysis for each cell supertype in each brain region to eliminate extremely sparse transcripts that could inflate model instability in the mixed‐effects framework.

NEBULA (negative binomial mixed model using large‐sample approximation) was used to identify DEGs at the supertype level and within each brain region through pairwise comparisons among the LATE‐NC, ADNC, and LATE‐NC&ADNC groups using the NEBULA R package v1.5.5.^46^ NEBULA is a statistical framework designed for differential expression analysis in multi‐subject single‐cell RNA‐seq data that accounts for both cell‐level and subject‐level variability.^42^, ^46^, ^47^ The NEBULA‐HL (hierarchical likelihood) method is optimized for estimating both cell‐level and donor‐level overdispersion. It fits a negative binomial mixed model that captures the hierarchical structure of single‐cell data by incorporating donor‐level random effects while modeling cell‐level variability. Genes detected in fewer than five cells were excluded using the NEBULA mincp parameter. Age at death, sex, PMI, self‐reported race, and apolipoprotein E genotype (ε2/ε2, ε2/ε3, ε2/ε4, ε3/ε3, ε3/ε4, and ε4/ε4) were included as covariates. The donor ID was used as a random effect. The total UMI count per nucleus was specified as an offset/scaling factor to account for differences in sequencing depth across cells. The supplementary metadata table (Table S3 in supporting information) contains the donor‐level covariates and random‐effect variable used in the models, and the Zenodo‐deposited scripts provide the NEBULA model for each supertype‐specific DEG analysis. Benjamini–Hochberg–adjusted *P* values were calculated to control the false discovery rate (FDR). A log_2_ fold‐change threshold of 0.58 (i.e., 1.5‐fold change) and adjusted *P* value < 0.05 were used to prioritize biologically meaningful transcriptional differences while reducing noise from small effect sizes. DEGs were annotated using the Alliance Genome Resource,^48^ which categorizes gene–disease associations based on high‐level Disease Ontology (DO) terms such as “Alzheimer's disease” and “dementia.” This approach allowed contextualization of disease‐relevant transcriptional patterns and examined how known disease‐associated genes are differentially regulated across LATE‐NC, ADNC, and LATE‐NC&ADNC.

#### 2.2.8 Biological pathway enrichment analysis

GSEA^49^ was applied to identify significant Kyoto Encyclopedia of Genes and Genomes (KEGG) and Gene Ontology (GO) pathways using the fgsea R package (v.1.32.4) ^49^ with the hallmark, KEGG, and GO gene set lists from MSigDB (msigdbr, v.24.1.0).^50^ For each cell supertype per group per brain region, genes were ranked for GSEA using the signed statistic derived from the NEBULA model (sign(log_2_fold change) × (–log_10_[adjusted *P* value]). Then the GSEA analysis was performed using the fgseaMultilevel function with default settings. FDR‐adjusted *P* values < 0.05 were considered significant KEGG or GO pathways. The R package ggplot2^51^ was used to generate the bar plots. Among significant pathways, enrichment strength and direction were evaluated using the normalized enrichment score (NES) in each cell supertype. The top 10 positively and 10 negatively enriched pathways were selected based on NES magnitude.

#### 2.2.9 Cell–cell communication analysis

Cell–cell communication analysis was performed using the R package CellChat (v2.2.0.9001) ^53^ to infer ligand–receptor interactions among non‐neuronal MTG cell supertypes on the same processed Seurat object used for the DEG analyses. Three separated CellChat objects were created using the normalized data matrix for the three neuropathological groups of interest: ADNC, LATE‐NC, and LATE‐NC&ADNC. Cell identities were defined using the SEA‐AD supertype annotations. To reduce noise from rare cell populations, we retained only cell supertypes represented by at least 50 cells in each of the three pathology groups. After filtering, cells were optionally downsampled within each pathology group and supertype, with a maximum of 2000 cells retained per group–supertype combination. This step was used to reduce the influence of highly abundant cell populations while preserving sufficient representation of each retained supertype. The CellChat human ligand‐receptor database was used for inference. Standard CellChat preprocessing was then performed, including subsetting to signaling genes, identifying overexpressed genes, and identifying overexpressed ligand‐receptor interactions. Expression values were smoothed using the human protein‐protein interaction network when supported by the installed CellChat version. Communication probabilities were inferred using the computeCommunProb() function, with population size considered and a trimmed mean threshold of 0.1. Weak communication events were removed using the filterCommunication() function with a minimum cell threshold of 10. Pathway‐level communication probabilities were computed with the computeCommunProbPathway() function, and aggregate communication networks were generated using the aggregateNet () function. Network centrality measures were then computed at the pathway level using the netAnalysis_computeCentrality() function. CellChat objects from the three pathology groups were merged for comparative analyses. We compared the total number and overall strength of inferred interactions across ADNC, LATE‐NC, and the LATE‐NC&ADNC. Signaling role analysis was used to identify cell supertypes with high outgoing interaction strength, interpreted as major signal senders, and high incoming interaction strength, interpreted as major signal receivers. Pairwise differential sender–receiver analyses were performed for ADNC versus LATE‐NC, ADNC versus LATE‐NC&ADNC, and LATE‐NC versus LATE‐NC&ADNC using differences in aggregate interaction strength. Circle plots and heatmaps were generated using the plotting functions in the CellChat to visualize global communication patterns and were included as supplementary visualizations.

## 3 RESULTS

### 3.1 Cohort characteristics across neuropathological groups

Figure 1B shows the age at death, sex, and PMI distribution of the four neuropathological groups. Age at death was not significantly different across neuropathological groups (Kruskal–Wallis *P* value = 0.084), whereas PMI differed significantly across groups (Kruskal–Wallis *P* value = 0.007). Sex distribution did not differ significantly across groups (Fisher exact test *P* value = 0.863). Post hoc pairwise Wilcoxon tests with Benjamini–Hochberg correction indicated that the PMI difference was driven by the comparison between the minimal‐to‐no ADNC/LATE‐NC and LATE‐NC groups (adjusted *P* value = 0.002). Because the minimal‐to‐no ADNC/LATE‐NC group was not included in downstream analyses, PMI was reassessed after excluding this group and was no longer statistically significant among the remaining groups (Kruskal–Wallis *P* value = 0.067). Similar results were observed in the A9 brain region.

### 3.2 Cell type annotation and data quality assessment in the MTG

**FIGURE 2:**
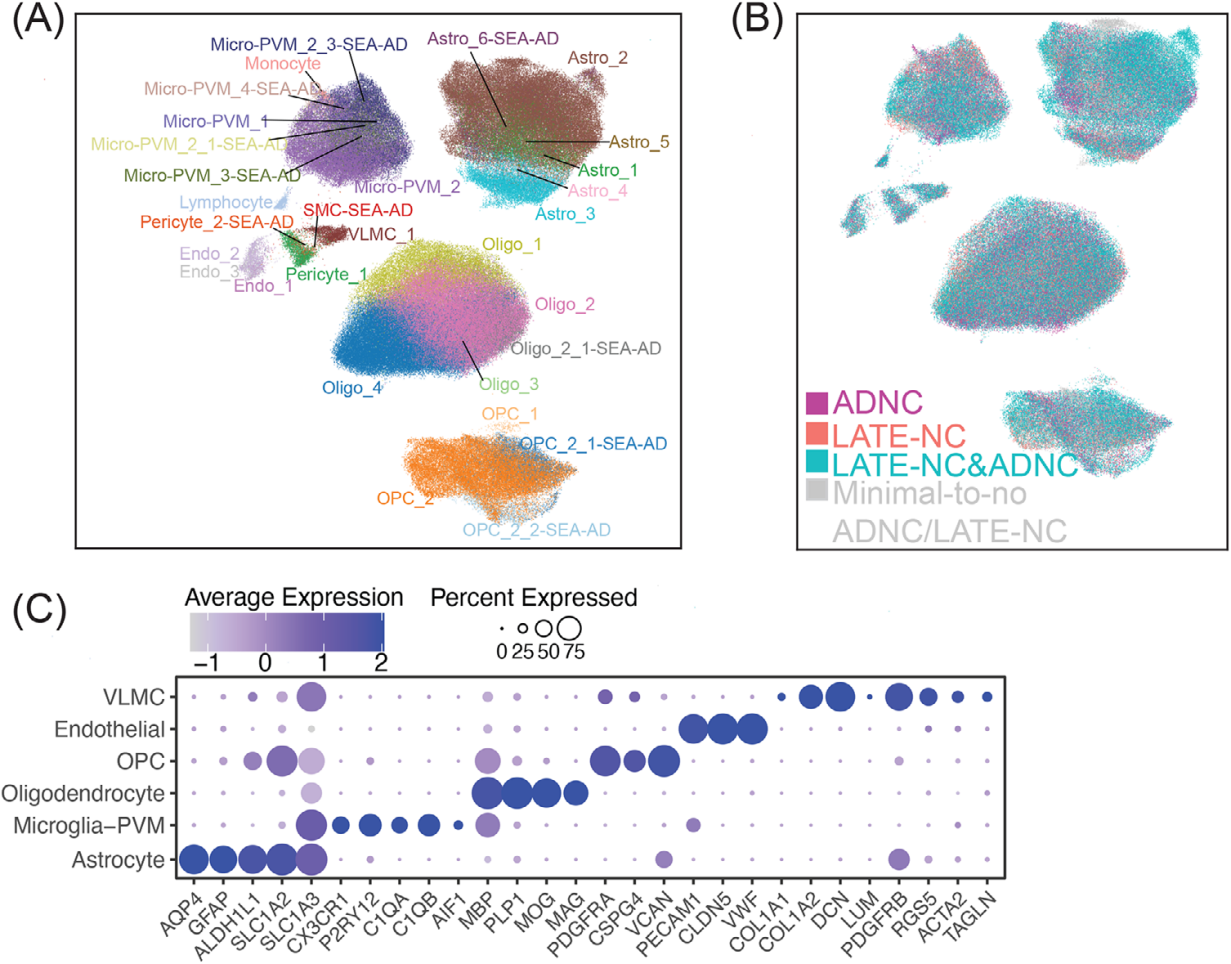
Cell supertype identification in the MTG brain region. A, UMAP visualization of cells colored by annotated supertypes in MTG. B, UMAP visualization of the same cells colored by disease groups (LATE‐NC, ADNC, and LATE‐NC&ADNC). C, Dot plot showing expression of canonical marker genes across major non‐neuronal subclasses. Dot size represents the percentage of cells expressing each marker, and color intensity represents the average scaled expression level. Canonical marker expression supports the transferred subclass annotations, including astrocytes, microglia/PVM, oligodendrocytes, OPCs, endothelial cells and VLMCs populations. ADNC, Alzheimer's disease neuropathologic change; LATE‐NC, limbic‐predominant age‐related transactive response DNA binding protein 43 kDa encephalopathy neuropathological change; MTG, middle temporal gyrus; OPC, oligodendrocyte precursor cell; PVM, perivascular macrophage; UMAP, Uniform Manifold Approximation and Projection; VLMC, vascular leptomeningeal cell.

Figure 2A shows the UMAP embedding of non‐neuronal cells from the MTG that was generated using the latent space provided in the SEA‐AD AnnData objects. Cells from all neuropathological groups were distributed across cell supertypes (Figure 2B), which indicates no strong grouping by neuropathological groups and minimal evidence of batch‐driven clustering. This supports the suitability of the MTG dataset for downstream differential expression analyses across different neuropathological groups. To investigate the accuracy transferred cell annotations, we first visualized canonical marker gene expression across major subclasses using dot plots (Figure 2C and Figure S2 in supporting information). Astrocytes, microglia/ perivascular macrophages (PVMs), oligodendrocytes, OPCs, endothelial cells, and VLMC/pericyte/smooth muscle‐associated cells showed the expected enrichment of canonical markers. Because subsequent differential expression analyses were performed at the supertype level, we further examined representative SEA‐AD–derived supertype marker genes using violin plots within each parent subclass. These plots were used to confirm that the supertypes included in our analyses showed marker‐expression patterns consistent with the SEA‐AD taxonomy. The overall distribution of cell types across conditions for the MTG region, UMAP visualizations, and cell‐type composition analyses for the A9 region are shown in Figure S2.

To assess donor‐level heterogeneity in cellular composition, we calculated the proportion of each non‐neuronal cell supertype for every donor and visualized these proportions using a donor‐level stacked bar plot ordered by neuropathologic group in Figure S3 in supporting information. We then tested whether cell‐supertype proportions differed by neuropathologic group using beta regression at the donor level. For each cell supertype, the beta‐regression model included neuropathologic group, age at death, sex, PMI, and total cell number per donor as covariates. We also used the same modeling framework to evaluate associations between cell‐supertype proportions and age at death. After Benjamini–Hochberg correction, no cell supertype showed a significant difference in proportion across neuropathologic groups, and no cell supertype showed a significant association with age at death. These findings suggest that group‐level differences in non‐neuronal cell‐supertype composition were not associated with neuropathologic diagnosis or age in the present cohort.

In the A9 region, age at death was not significantly associated with donor‐level cell‐supertype proportions after Benjamini–Hochberg correction. In contrast, the global beta‐regression test identified significant group‐associated differences for Oligo_2 and OPC_1 after Benjamini–Hochberg correction. However, in post hoc pairwise comparisons, only Oligo_2 showed a pairwise contrast that remained significant after multiple‐testing correction, primarily driven by the difference between the ADNC and LATE‐NC&AD groups. OPC_1 showed a significant global group effect, but no individual pairwise contrast survived correction (Figure S3).

We then evaluated the expression of canonical neuronal marker genes across all non‐neuronal cell classes to assess potential neuronal RNA contamination. Neuronal markers were strongly enriched in neuronal populations, while expression in non‐neuronal classes remained low (generally < 5% of neuronal levels, Figure S4 in supporting information for MTG, Figure S5 in supporting information for A9, and Table S4 in supporting information).

### 3.3 Differential gene expression analysis

**FIGURE 3:**
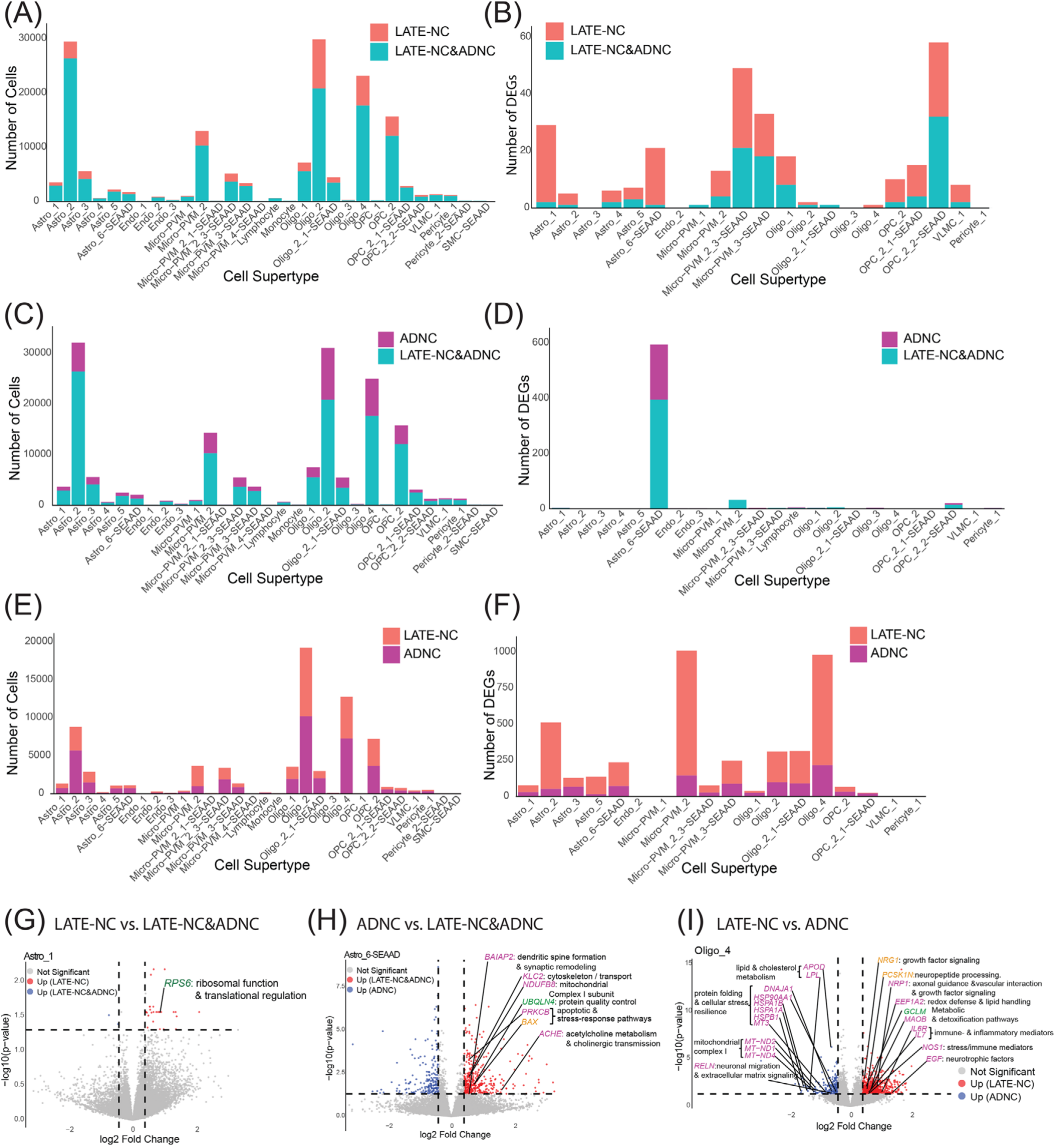
Cell‐supertype abundance and differential gene expression across neuropathological groups in the MTG region. A, Number of cells per non‐neuronal cell supertype for LATE‐NC versus LATE‐NC&ADNC. B, Number of DEGs identified per cell supertype for LATE‐NC versus LATE‐NC&ADNC. C, Number of cells for ADNC versus LATE‐NC&ADNC. D, Number of DEGs for ADNC versus LATE‐NC&ADNC. E, Number of cells for LATE‐NC versus ADNC. F, Number of DEGs for LATE‐NC versus ADNC. G–I, Representative volcano plots showing DEGs for selected cell supertypes in the LATE‐NC versus LATE‐NC&ADNC, ADNC versus LATE‐NC&ADNC, and LATE‐NC versus ADNC comparisons, respectively. In each volcano plot, significant genes upregulated in the first group listed in the comparison are shown in red, significant genes upregulated in the second group are shown in blue, and non‐significant genes are shown in gray. Dashed lines indicate significance thresholds: the horizontal line represents an adjusted *P* value of 0.05, and vertical lines represent a fold‐change threshold of 1.5. Annotated genes are colored according to disease relevance based on the Alliance of Genome Resources:^48^ purple, AD‐related genes; green, dementia‐related genes; orange, genes associated with both AD and dementia. AD, Alzheimer's disease; ADNC, Alzheimer's disease neuropathologic change; LATE‐NC, limbic‐predominant age‐related transactive response DNA binding protein 43 kDa encephalopathy neuropathological change; MTG, middle temporal gyrus.

We examined non‐neuronal cell‐supertype distributions in the MTG across neuropathologic comparisons (Figure 3A–F). Across comparisons, astrocytes and oligodendrocytes were the dominant populations, while microglia, OPCs, endothelial cells, VLMCs, and pericytes were less abundant. The LATE‐NC&ADNC group contributed more cells than the single‐pathology groups, which reflects the larger number of mixed‐pathology donors.

The number of DEGs was highly cell type and comparison specific. In LATE‐NC versus LATE‐NC&ADNC, the strongest DEG patterns were observed in OPC_2_2‐SEAAD, with additional differences in astrocytic and disease‐associated microglial populations (Figure 3B). In ADNC versus LATE‐NC&ADNC, DEG patterns were more restricted, with the clearest signal in Astro_6‐SEAAD and limited changes across most other populations (Figure 3D). The direct LATE‐NC versus ADNC comparison showed broader transcriptional divergence, particularly in microglial and oligodendrocyte populations, with a higher number of DEGs observed in LATE‐NC (Figure 3F). Additional volcano plots for DEG results in the MTG region are provided in Figure S6 in supporting information. The full list of DEGs and the corresponding analyses in the A9 region are provided in the supporting information (Figure S7 for LATE‐NC vs. LATE‐NC&ADNC, Figure S8 for ADNC vs. LATE‐NC&ADNC, Figure S9 for LATE‐NC vs. ADNC and Tables S5–S7).

#### 3.3.1 LATE‐NC versus LATE‐NC&ADNC

In the comparison between LATE‐NC and mixed LATE‐NC&ADNC, selected DEGs suggested cell type–specific transcriptional differences between isolated LATE‐NC and concomitant pathology (Table 2, Figure 3G). In astrocyte populations, LATE‐NC showed upregulation of representative genes related to translational regulation and redox defense, including *RPS6* in Astro_1 and *GSTM3* in Astro_5. In contrast, LATE‐NC&ADNC showed upregulation of genes related to cytoskeletal regulation and mitochondrial function in microglial and OPC populations, including *BAIAP2* in Micro‐PVM_2_3‐SEAAD and *NDUFB8* in OPC_2_2‐SEAAD. Detailed gene‐level interpretations are provided in Table 2. Comparable but quantitatively distinct transcriptional patterns were observed in the A9 region, where LATE‐NC generally exhibited a higher DEG burden than LATE‐NC&ADNC, particularly in astrocytic populations.

**TABLE 2:** Genes differentially expressed in glial and vascular cell supertypes from the middle temporal gyrus in the Seattle Alzheimer's Disease Brain Cell Atlas (SEA‐AD) consortium LATE‐NC, ADNC, and mixed pathology (LATE‐NC&ADNC) groups.

Cell supertype	Gene	Upregulated group	Interpretation/comment	Cite
**LATE‐NC—LATE‐NC&ADNC pairwise comparison**				
Astro_1	*RPS6*	LATE‐NC	Suggests selective activation of ribosomal and translational control programs in LATE‐NC relative to LATE‐NC&ADNC.	^53^
Astro_5	*GSTM3*	LATE‐NC	Suggests enhanced astrocyte‐mediated redox responses in LATE‐NC pathology.	^54^
Micro‐PVM_2_3‐SEAAD	*BAIAP2*	LATE‐NC&ADNC	Gene is a regulator of cytoskeletal dynamics and neuroinflammatory signaling.	^55^
OPC_2_2‐SEAAD	*NDUFB8* *SQSTM1*	LATE‐NC&ADNC	Consistent with mitochondrial dysfunction characteristic of ADNC and related pathology. Consistent with increased proteostatic stress and impaired clearance of misfolded proteins in neurodegenerative disease	^56^, ^57^
**ADNC—LATE‐NC&ADNC pairwise comparison**				
Astro_1	*ACADVL*	LATE‐NC&ADNC	Previously implicated in AD through its role in mitochondrial fatty acid metabolism.	^58^
Astro_1	*NDUFC2*	LATE‐NC&ADNC	Gene encodes a nuclear subunit of mitochondrial complex I; it has been associated with mitochondrial dysfunction.	^59^
Astro_6‐SEAAD	*BAIAP2, PRKCB, KLC2*	LATE‐NC&ADNC	Genes are linked to cytoskeletal remodeling, intracellular signaling, and axonal transport processes, respectively, suggesting altered cellular structural dynamics and signaling regulation under mixed pathology.	^55^, ^59^, ^60^
Astro_6‐SEAAD	*BAX*	LATE‐NC&ADNC	Gene is a pro‐apoptotic regulator which has been implicated in AD and broader dementia‐related neurodegeneration.	^61^
Lymphocytes	*PRKN*	LATE‐NC&ADNC	Consistent with altered mitochondrial quality control and neurodegenerative processes.	^59^
Oligo_1	*NGFR*	LATE‐NC&ADNC	Neurotrophic signaling and cellular stress response genes that involved in AD pathogenesis.	^59^, ^62^, ^63^, ^64^
**LATE‐NC—ADNC pairwise comparison**				
Astro_1	*PAK1, FGFR1*	LATE‐NC	Genes are related to intracellular signaling and growth factor–associated regulatory pathways, suggesting altered astrocytic signaling and adaptive cellular responses in LATE relative to AD.	^65^, ^66^
Astro_6‐SEAAD	*LDLR, ASS1, MT1F*	LATE‐NC	Genes are involved in lipid/cholesterol metabolism, amino acid metabolism, metal homeostasis, and stress‐response pathways, suggesting broader metabolic and protective changes in LATE astrocytes.	^67^, ^68^, ^69^
Astro_2 & Astro_3	*MT‐ND1, MT‐ND2, MT‐ND4*	ADNC	Mitochondrial‐encoded complex I genes were upregulated, suggesting increased oxidative phosphorylation and mitochondrial activity in AD astrocytes.	^70^, ^71^, ^72^
Astro_1 & Astro_6‐SEAAD	*ALDH2, NOTCH2*	ADNC	Genes are associated with oxidative stress mitigation and astrocytic signaling/cell‐fate regulation, suggesting stress‐related and signaling changes in AD astrocytes.	^69^, ^73^
Micro‐PVM_2	*HMGCR, SREBF2, CYP51A1, LDLR*	LATE‐NC	Genes are involved in sterol, lipid, and cholesterol metabolism, suggesting active metabolic regulation in LATE microglia.	^68^, ^69^, ^74^, ^75^, ^76^, ^77^, ^78^
Micro‐PVM_2	*SERPINF1, SERPINE2, PLAU*	LATE‐NC	Genes are associated with extracellular matrix remodeling, protease regulation, and tissue‐response pathways, suggesting altered microglial involvement in local remodeling processes.	^69^, ^79^, ^80^, ^81^, ^82^
Micro‐PVM_2 & Micro‐PVM_3‐SEAAD	*CDK5, PTGS1, NDUFA2, SLC8A3*	LATE‐NC	Genes are involved in stress response, inflammatory/metabolic regulation, mitochondrial function, and ion homeostasis, suggesting active cellular regulation in LATE microglia.	^69^, ^83^, ^84^, ^85^, ^86^
Micro‐PVM_2 & Micro‐PVM_2_3‐SEAAD & Micro‐PVM_3‐SEAAD	*MT‐ND1, MT‐ND2, MT‐ND4*	ADNC	Mitochondrial complex I genes were upregulated, suggesting mitochondrial hyperactivity or altered oxidative phosphorylation in AD microglia.	^70^, ^71^, ^72^
Micro‐PVM_2 & Micro‐PVM_2_3‐SEAAD	*IRS2, LRRK2*	ADNC	Genes are associated with insulin signaling and kinase‐mediated inflammatory/stress pathways, suggesting pathogenic signaling changes in AD microglia.	^87^, ^88^
Oligo_2_1‐SEAAD & Oligo_4	*PAK1, NRG1, NRP1, EGF*	LATE‐NC	Genes are related to growth factor signaling, intracellular signaling, and cell–cell communication, suggesting active regulatory and adaptive signaling programs in LATE oligodendrocytes.	^65^, ^69^, ^89^, ^90^, ^91^, ^92^, ^93^, ^94^, ^95^
Oligo_2_1‐SEAAD & Oligo_4	*TLR4, IL7, IL6R*	LATE‐NC	Immune‐ and inflammatory‐related genes were upregulated, suggesting enhanced neuroimmune crosstalk and inflammatory signaling regulation in LATE oligodendrocytes.	^96^, ^97^, ^98^, ^99^
Oligo_2 & Oligo_4	*GSTM3, FABP3, GCLM, MAOB, EEF1A2*	LATE‐NC	Genes are involved in detoxification, lipid handling, redox defense, metabolic regulation, and protein translation, suggesting stress‐adaptive and metabolic support functions in LATE oligodendrocytes.	^54^, ^100^, ^101^, ^102^, ^103^, ^104^
Oligo_4	*PCSK1N*	LATE‐NC	Gene is associated with neuropeptide processing–related pathways, suggesting altered regulatory functions in LATE oligodendrocytes.	^105^
Oligo_1 & Oligo_4	*MT‐ND1, MT‐ND2, MT‐ND4, NDUFA2*	ADNC	Mitochondrial complex I–related genes were upregulated, suggesting increased mitochondrial activity and metabolic stress in AD oligodendrocytes.	^70^, ^71^, ^72^, ^84^
Oligo_2 & Oligo_4	*LPL, APOD, MT3*	ADNC	Genes are associated with lipid metabolism, lipid transport, and metal‐binding/stress‐response pathways, suggesting lipid dysregulation and stress‐related metabolic changes in AD oligodendrocytes.	^106^, ^107^, ^108^, ^109^, ^110^, ^111^, ^112^, ^113^, ^114^
Oligo_4	*DNAJA1, DNAJA4, HSP90AA1, HSPA1A, HSPA1B, HSPB1*	ADNC	Chaperone and heat‐shock genes were strongly upregulated, indicating heightened proteostatic stress in AD oligodendrocytes.	^69^, ^115^, ^116^, ^117^
Oligo_2 & Oligo_4	*EGFR, BCL2, RELN*	ADNC	Genes are associated with growth factor signaling, cell survival, and extracellular signaling, suggesting altered survival and regulatory pathways in AD oligodendrocytes.	^69^, ^118^, ^119^, ^120^, ^121^
OPC_2	*NRP1*	LATE‐NC	Upregulation of *NRP1* suggests preserved growth factor signaling, vascular interaction, and supportive communication capacity in LATE OPCs.	^92^
OPC_2__1‐SEAAD	*MT3, APOD*	ADNC	Genes are associated with metal‐binding responses and lipid transport, suggesting stress‐related metabolic imbalance in AD OPCs.	^106^, ^107^, ^109^, ^110^

Abbreviations: AD, Alzheimer's disease; ADNC, Alzheimer's disease neuropathologic change‐dominant group; Astro_1, astrocyte supertype 1; Astro_2, astrocyte supertype 2; Astro_3, astrocyte supertype 3; Astro_5, astrocyte supertype 5; Astro_6‐SEAAD, astrocyte supertype 6, SEA‑AD–associated astrocytic state; Cite, citation supporting Interpretation/comment; LATE‐NC, limbic‐predominant age‐related transactive response DNA binding protein 43 kDa encephalopathy neuropathological change; LATE‐NC&ADNC, mixed LATE‐NC and ADNC group; Micro‐PVM_2, microglia/perivascular macrophage–like supertype 2; Micro‐PVM_2_3‐SEAAD, microglia–PVM intermediate supertype (clusters 2_3), SEA‑AD–associated; Micro‐PVM_3‐SEAAD, Microglia / perivascular macrophage–like supertype 3, SEA‑AD–associated; Oligo_1, Oligodendrocyte supertype 1; Oligo_2, oligodendrocyte supertype 2; Oligo_2_1‐SEAAD, oligodendrocyte supertype 2_1 intermediate, SEA‑AD–associated; Oligo_4, oligodendrocyte supertype 4; OPC_2, oligodendrocyte precursor cell supertype 2; OPC_2_1‐SEAAD, OPC supertype 2_1 intermediate, SEA‑AD–associated; OPC_2_2‐SEAAD, OPC supertype 2_2, SEA‑AD–associated.

#### 3.3.2 ADNC versus LATE‐NC&ADNC

Comparison of ADNC with LATE‐NC&ADNC identified selected DEGs that may reflect molecular features associated with mixed pathology (Table 2, Figure 3H). In Astro_1, LATE‐NC&ADNC showed upregulation of *ACADVL* and *NDUFC2*, genes related to mitochondrial fatty acid metabolism and mitochondrial complex I function. In Astro_6‐SEAAD, upregulated genes in LATE‐NC&ADNC included *BAIAP2, PRKCB, KLC2*, and *BAX*, which are associated with cytoskeletal regulation, intracellular signaling, axonal transport, and apoptosis‐related processes. Additional upregulated genes in lymphocyte and oligodendrocyte populations, including *PRKN* and *NGFR*, further suggest cell type–specific changes related to mitochondrial QC and cellular stress responses.

#### 3.3.3 LATE‐NC versus ADNC

The direct comparison between LATE‐NC and ADNC revealed distinct gene‐level signatures across glial populations (Table 2, Figure 3I). In LATE‐NC, selected DEGs included genes associated with growth factor signaling, lipid and cholesterol regulation, extracellular remodeling, immune‐related regulation, redox balance, and cellular stress adaptation. These patterns were observed across astrocyte, microglial, oligodendrocyte, and OPC populations and suggest that LATE‐NC may be associated with adaptive regulatory and metabolic gene expression changes.

In contrast, ADNC showed upregulation of selected genes related to mitochondrial complex I function, heat‐shock and chaperone activity, lipid transport, metal‐binding stress responses, and apoptosis‐ or survival‐associated regulation. These changes were particularly evident in microglial and oligodendrocyte populations and suggest stronger mitochondrial and proteostatic stress‐related gene expression in ADNC. Thus, at the DEG level, LATE‐NC and ADNC appear to show different glial gene‐expression patterns, with LATE‐NC showing more regulatory and stress‐adaptive features and ADNC showing stronger mitochondrial and proteostatic stress‐associated features.

### 3.4 Enrichment analysis

**FIGURE 4:**
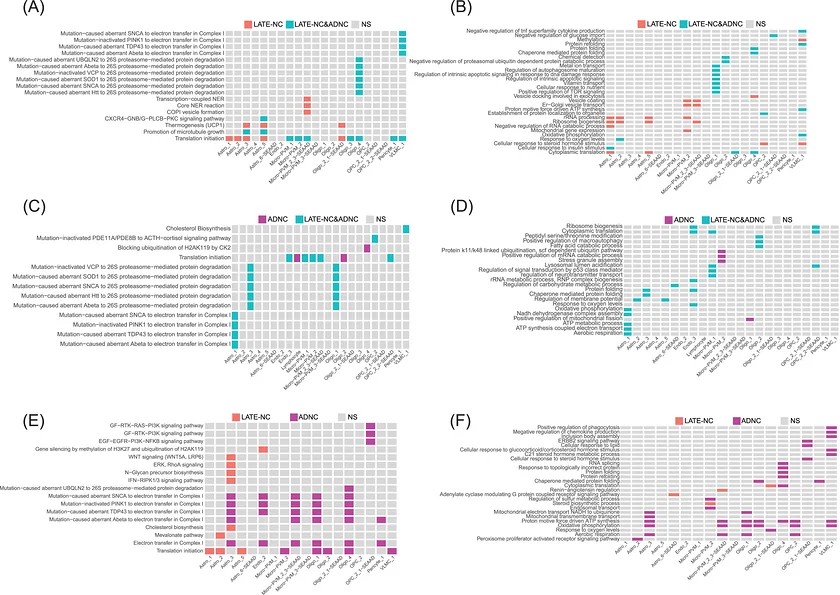
KEGG Pathway and GO enrichment across neuropathological group comparisons in the MTG region. Heatmaps summarize significantly enriched KEGG pathways and GO biological process terms across non‐neuronal cell supertypes in the MTG region. A, B, Enrichment results for LATE‐NC versus LATE‐NC&ADNC, with pathway enrichment shown in (A) and GO term enrichment shown in (B). C, D, Enrichment results for ADNC versus LATE‐NC&ADNC, with pathway enrichment shown in (C) and GO term enrichment shown in (D). E, F, Enrichment results for LATE‐NC versus ADNC, with pathway enrichment shown in (E) and GO term enrichment shown in (F). Colored squares indicate significant enrichment in the corresponding neuropathological group, whereas gray squares indicate non‐significant enrichment. Group colors correspond to those used throughout the manuscript: LATE‐NC, salmon; ADNC, purple; and LATE‐NC&ADNC, teal. ADNC, Alzheimer's disease neuropathologic change; GO, Gene Ontology; KEGG, Kyoto Encyclopedia of Genes and Genomes; LATE‐NC, limbic‐predominant age‐related transactive response DNA binding protein 43 kDa encephalopathy neuropathological change; MTG, middle temporal gyrus.

Enriched KEGG and GO pathways are summarized qualitatively across cell supertypes in Figure 4A–F, as NESs are not directly comparable across conditions due to gene filtering steps. Detailed KEGG and GO annotations, directionality, and interpretation are provided in Table 3. The full lists of KEGG and GO terms are available in Tables S8 through S10 in supporting information. Selected bar plots showing representative KEGG pathways and GO terms in the MTG region are provided in Figure S10 in supporting information. Corresponding analyses in the A9 region are provided in the supporting information (Figure S11 for LATE‐NC vs. LATE‐NC&ADNC, Figure S12 for ADNC vs. LATE‐NC&ADNC, Figure S13 for LATE‐NC vs. ADNC, and Tables S11–S13).

**TABLE 3:** Gene set enrichment analysis in glial and vascular cell supertypes from the middle temporal gyrus in the Seattle Alzheimer's Disease Brain Cell Atlas (SEA‐AD) consortium LATE‐NC, ADNC, and mixed pathology (LATE‐NC&ADNC) groups.

Cell class/supertype	Enriched group	Enriched pathways	Interpretation/comment	Cite
**LATE‐NC vs. LATE‐NC&ADNC: KEGG**				
Across all MTG cell populations	LATE‐NC and LATE‐NC&ADNC	Translation initiation	Consistent with altered protein synthesis programs reported in neurodegenerative disorders, including AD.	^122^
Astrocytes and microglia populations	LATE‐NC	Genome maintenance, metabolic adaptation, intracellular trafficking (including thermogenesis, nucleotide excision repair, COPI‐mediated vesicle formation)	DNA repair is an emerging hallmark of aging and AD, especially in microglia, suggests activation of genome maintenance mechanisms in LATE pathology. COPI vesicles playing a significant role in disruptions in protein trafficking between the Golgi and ER, which are related to both amyloid precursor protein (APP) processing and TDP‐43 mislocalization.	^123^, ^124^, ^125^, ^126^, ^127^, ^128^
Astrocytes	LATE‐NC&ADNC	Cytoskeletal remodeling, chemokine signaling	Suggests reactive astrocyte activation and inflammatory signaling.	^129^
Oligodendrocytes	LATE‐NC&ADNC	Engagement of proteasome‐mediated degradation pathways	Pathways include components previously linked to TDP‐43–associated neurodegeneration, which may reflect compensatory responses to increased protein misfolding and aggregation.	^130^
Vascular‐associated cell types	LATE‐NC&ADNC	Mitochondrial complex I electron transport, oxidative phosphorylation–related pathways	Consistent with mitochondrial dysfunction, oxidative stress, and blood–brain barrier vulnerability reported in neurodegenerative disease.	^131^, ^132^
**LATE‐NC vs. LATE‐NC&ADNC: GO**				
Astrocytic and oligodendrocytic populations	LATE‐NC (astrocytes) LATE‐NC&ADNC (oligodendrocytes)	Cytoplasmic translation and related ribosome‐associated processes	Consistent with altered protein synthesis programs observed in neurodegeneration.	^133^
Astrocyte	LATE‐NC	Ribosome biogenesis, rRNA processing	Processes previously implicated in AD but not yet well characterized in LATE‐NC.	^134^
Astrocyte	LATE‐NC	RNA metabolism, vesicular trafficking (including enrichment for negative regulation of RNA catabolism)	Process is mechanistically relevant to TDP‐43 biology and its autoregulatory feedback mechanisms.	^135^
Microglia	LATE‐NC	ER–Golgi vesicle transport and vesicle coating	Consistent with known roles of secretory and trafficking pathways in protein misprocessing and TDP‐43 mislocalization.	^126^
Astrocytes	LATE‐NC&ADNC	Metabolic stress, hormone signaling	The impairment of these pathways can lead to protein aggregation, neuroinflammation, and cognitive decline.	^136^, ^137^, ^138^, ^139^
Oligodendrocytes, vascular‐associated populations	LATE‐NC&ADNC	Multiple protein folding and chaperone‐associated processes	Consistent with canonical AD‐associated stress responses.	^137^, ^140^, ^141^, ^142^, ^143^
**ADNC vs. LATE‐NC&ADNC: KEGG**				
Lymphocytes and Oligo_2	ADNC	Translation initiation	Consistent with altered protein synthesis programs reported in neurodegenerative disorders, including AD.	^122^
Oligo_4	ADNC	CK2‐mediated regulation of histone H2AK119 ubiquitination	Both CK2 activity and H2AK119ub1 have been implicated in AD‐related neuroinflammation, tau pathology, and cognitive decline, although their direct mechanistic interaction remains incompletely defined.	^144^, ^145^
Microglia, OPCs, and oligodendrocytes	LATE‐NC&ADNC	Translation initiation	Consistent with dysregulated protein synthesis in mixed pathology.	^122^
Oligodendrocytes	LATE‐NC&ADNC	26S proteasome–mediated degradation of aberrant proteins	Suggests heightened proteostasis demand in the presence of combined disease burden.	^146^, ^147^, ^148^
Astrocytes	LATE‐NC&ADNC	Mitochondrial dysfunction–related pathways (including perturbations of electron transport chain Complex I involving Aβ‐, TDP‐43–, *PINK1*‐, *SNCA*‐associated mechanisms)	Consistent with oxidative stress and mitochondrial vulnerability in AD.	^131^, ^132^
**ADNC vs. LATE‐NC&ADNC: GO**				
Astro_1	LATE‐NC&ADNC	Mitochondrial respiration–related processes (including aerobic respiration, oxidative phosphorylation, ATP synthesis, NADH dehydrogenase complex assembly)	Pathways are tightly linked to AD‐associated bioenergetic failure and oxidative stress.	^149^
Astro_2, Astro_3, Astro_5, Astro_6‐SEAAD	LATE‐NC&ADNC	Regulation of membrane potential, carbohydrate metabolism, protein folding	Reflects combined metabolic and proteostatic stress associated with amyloid, tau, and TDP‐43–related pathology.	^130^, ^150^
Micro‐PVM_1	LATE‐NC&ADNC	Neurotransmitter transport, lysosomal lumen acidification processes	Consistent with altered metabolic and degradative activity accompanying AD pathology.	^151^
Microglia	ADNC	Enrichment of stress granule assembly, mRNA metabolism, ubiquitin‐dependent proteolysis pathways	Highlights perturbations in RNA handling and proteostasis even in the absence of overt LATE pathology.	^152^, ^153^
Oligodendrocytes	ADNC	Mitochondrial fission	Consistent with altered mitochondrial dynamics.	^154^
Oligodendrocytes	LATE‐NC&ADNC	Fatty acid catabolism, macroautophagy, phosphorylation‐mediated signaling	Suggests compensatory metabolic and stress‐response mechanisms under mixed pathology.	^155^, ^156^, ^157^
**LATE‐NC vs. ADNC: KEGG**				
Astrocytes	LATE‐NC	Translation initiation	Consistent with altered protein synthesis programs reported in neurodegenerative disorders, including AD.	^122^
Microglia, oligodendrocytes, and OPCs	ADNC	Translation initiation	Consistent with altered protein synthesis programs reported in neurodegenerative disorders, including AD.	^122^
Astro_2	LATE‐NC	Mevalonate pathway	Implicates altered production of farnesyl and geranylgeranyl pyrophosphate. This is the key intermediate for protein prenylation, the dysregulation of which may contribute to tau phosphorylation, neuronal dysfunction, and misfolded protein accumulation in both ADNC and LATE‐NC.	^158^, ^159^, ^160^, ^161^
Astro_3	LATE‐NC	Cholesterol biosynthesis	Pathway is known to regulate Aβ production and aggregation in AD but its role in LATE‐NC remains under investigation.	^162^, ^163^
Astrocytes	LATE‐NC	Inflammatory and signaling pathways (including IFN–RIPK1/3 signaling, ERK/RhoA signaling, WNT signaling)	These pathways have been linked to neuroinflammation, necroptosis, synaptic loss, and disease progression in AD. Emerging evidence suggests that TDP‐43 dysfunction may modulate IFN and RhoA/WNT signaling cascades.	^164^, ^165^, ^166^, ^167^, ^168^, ^169^
Nearly all non‐neuronal cell types, including astrocytes, endothelial cells, microglia, oligodendrocytes, and OPCs	ADNC	Mitochondrial dysfunction (including electron transport chain complex I driven by Aβ, TDP‐43, SNCA, PINK1 perturbations)	Reflects canonical AD‐associated oxidative stress and bioenergetic failure.	^170^, ^171^, ^172^, ^173^, ^174^, ^175^, ^176^
OPCs	ADNC	Growth factor–mediated signaling pathways (GF–RTK–RAS–PI3K and EGF–EGFR–PI3K–NFκB)	Pathways are known to promote neuroinflammation, amyloid deposition, and tau hyperphosphorylation.	^177^
**LATE‐NC vs. ADNC: GO**				
Astro_3, Oligo_1	ADNC	Mitochondrial transmembrane transport, NADH‐to‐ubiquinone electron transport	Supports widespread disruption of mitochondrial homeostasis across glial lineages.	^178^, ^179^, ^180^
Oligodendrocytes, vascular‐associated cells	ADNC	Chaperone‐mediated folding, protein refolding, responses to misfolded proteins	These pathways play a critical role in mitigating protein misfolding by facilitating the degradation of pathological aggregates such as tau and TDP‐43.	^141^, ^142^, ^143^, ^150^, ^181^, ^182^, ^183^, ^184^
Oligodendrocytes	ADNC	RNA splicing	Consistent with RNA dysregulation linked to both AD and TDP‐43 biology.	^185^, ^186^, ^187^
Astrocytes, microglia	LATE‐NC	GPCR‐linked signaling programs such as adenylate cyclase–modulating G‐protein–coupled receptor signaling, renin–angiotensin regulation	Such pathways have been linked to neuroinflammatory signaling, oxidative stress responses, and regulation of cerebral blood flow and metabolic homeostasis in neurodegeneration.	^188^, ^189^, ^190^, ^191^, ^192^
Micro‐PVM_2	LATE‐NC	Lipid‐ and steroid‐related metabolic processes (including enrichment of steroid biosynthetic pathways)	Consistent with reported links between sterol metabolism, neurodegeneration, and regulatory effects associated with TDP‐43–related mechanisms.	^193^, ^194^
Micro‐PVM_2	ADNC	Endosomal transport, sulfur metabolic processes	Pathways relevant to amyloid/tau handling and oxidative‐inflammatory stress.	^195^, ^196^
VLMCs	ADNC	Immune‐regulatory programs	The dysregulation or failure of chemokine production can lead to a persistent inflammatory state that worsens AD progression. The dysregulation of phagocytosis can lead to the accumulation of Aβ plaques and increased neuroinflammation in AD.	^197^, ^198^, ^199^, ^200^
Oligodendrocytes	LATE‐NC ADNC	Shared cytoplasmic translation	Has been reported to be directly related to protein synthesis.	^133^, ^201^

Abbreviations: Aβ, amyloid beta; AD, Alzheimer's disease; ADNC, Alzheimer's disease neuropathologic change‐dominant group; Astro_1, astrocyte supertype 1; Astro_2, astrocyte supertype 2; Astro_3, astrocyte supertype 3; Astro_5, astrocyte supertype 5; Astro_6‐SEAAD, astrocyte supertype 6, SEA‑AD–associated astrocytic state; ATP, adenosine triphosphate; Cite, citation supporting Interpretation/comment; ER, endoplasmic reticulum; GO, Gene Ontology; GPCR, G protein‐coupled receptor; KEGG, Kyoto Encyclopedia of Genes and Genomes; LATE‐NC&ADNC, mixed LATE‐NC and ADNC group; LATE‐NC, limbic‐predominant age‐related transactive response DNA binding protein 43 kDa encephalopathy neuropathological change; Micro‐PVM_1, Microglia/perivascular macrophage–like supertype 1; Micro‐PVM_2, Microglia/perivascular macrophage–like supertype 2; MTG, middle temporal gyrus; NADH, nicotinamide adenine dinucleotide + hydrogen; Oligo_1, oligodendrocyte supertype 1; Oligo_2, oligodendrocyte supertype 2; Oligo_4, oligodendrocyte supertype 4; OPC, oligodendrocyte precursor cell; TDP‐43, transactive response DNA binding protein 43 kDa; VLMC, vascular leptomeningeal cell.

#### 3.4.1 Functional pathway differences between LATE‐NC and LATE‐NC&ADNC in MTG

In KEGG enrichment analysis across MTG non‐neuronal cell populations, both LATE‐NC and LATE‐NC&ADNC showed enrichment of pathways related to translation initiation, consistent with altered protein synthesis programs previously reported in neurodegenerative disorders, including AD^122^ (Figure 4A). However, the enrichment patterns differed between the two neuropathologic groups (Table 3).

Pathway enrichment analysis identified both shared and pathology‐specific patterns between LATE‐NC and LATE‐NC&ADNC (Figure 4A, Table 3). Pathways related to translation initiation were observed in both groups, while additional enriched terms differed by pathological context. In LATE‐NC, enriched pathways included categories related to genome maintenance, metabolic regulation, and intracellular trafficking. In LATE‐NC&ADNC, enriched pathways included proteostasis‐related terms, translational regulation, inflammatory signaling, and mitochondrial function.

These enrichment patterns varied across cell supertypes (Table 3). Microglial populations in LATE‐NC showed enrichment of genome maintenance‐related pathways, whereas astrocytic populations in LATE‐NC&ADNC showed enrichment of cytoskeletal and chemokine‐related pathways. Oligodendrocyte populations in LATE‐NC&ADNC showed enrichment of proteasome‐associated pathways, and vascular‐associated cell types showed enrichment of mitochondrial electron transport and oxidative phosphorylation‐related terms.

Consistent with the KEGG results, GO terms related to cytoplasmic translation and ribosome‐associated processes were enriched across astrocytic and oligodendrocytic populations, supporting altered protein synthesis programs in both LATE‐NC and LATE‐NC&ADNC (Table 3, Figure 4B). In LATE‐NC, enriched GO terms were related to ribosome biogenesis, rRNA processing, RNA metabolism, and vesicular trafficking, including negative regulation of RNA catabolism in astrocytes and endoplasmic reticulum–Golgi vesicle transport in microglial populations (Table 3). These findings are consistent with RNA‐processing and trafficking‐related mechanisms relevant to TDP‐43 biology. In contrast, LATE‐NC&ADNC showed broader enrichment of GO terms related to metabolic stress, protein folding, and chaperone‐associated processes, particularly in oligodendrocyte and vascular‐associated populations.

Overall, the KEGG and GO results showed overlapping enrichment of translation‐ and proteostasis‐related biology, while GO analysis provided additional resolution of RNA metabolism, vesicular trafficking, and protein‐folding processes across cell supertypes.

#### 3.4.2 Functional pathway differences between ADNC and LATE‐NC&ADNC in MTG

In a KEGG enrichment analysis, in the ADNC group, enriched pathways were limited to a small number of cell populations and included translation initiation in lymphocytes and Oligo_2 cells, as well as CK2‐mediated regulation of histone H2AK119 ubiquitination in Oligo_4 (Figure 4C, Table 3).

In contrast, the LATE‐NC&ADNC group showed broader enrichment of translation initiation pathways across microglial, OPC, and oligodendrocyte populations (Table 3). Mixed pathology was also associated with enrichment of proteasome‐mediated degradation pathways in oligodendrocytes and mitochondrial dysfunction‐related pathways in astrocytes, including electron transport chain Complex I‐related terms.

GO terms in the LATE‐NC&ADNC group highlighted mitochondrial respiration, oxidative phosphorylation, translation, and proteostasis‐related processes across multiple glial populations, which is consistent with the KEGG results (Table 3, Figure 4D).

Astrocytic populations showed the clearest GO‐level divergence in the LATE‐NC&ADNC group, which is associated with broader bioenergetic and proteostatic stress–related processes (Table 3). The microglial populations differed by condition, with LATE‐NC&ADNC showing enrichment of lysosomal and transport‐related processes, whereas ADNC showed enrichment of RNA metabolism, stress granule assembly, and ubiquitin‐dependent proteolysis. Oligodendrocyte and OPC populations further supported condition‐specific differences, with ADNC showing enrichment of mitochondrial dynamics‐related terms and LATE‐NC&ADNC showing enrichment of fatty acid catabolism, macroautophagy, phosphorylation‐related signaling, ribosome biogenesis, and cytoplasmic translation (Table 3).

Overall, KEGG and GO analyses indicate that LATE‐NC&ADNC showed stronger enrichment of bioenergetic, metabolic, translational, proteostatic, and autophagy‐related processes, whereas ADNC showed more restricted enrichment related to RNA metabolism, ubiquitination, and mitochondrial dynamics.

#### 3.4.3 Functional pathway differences between LATE‐NC and ADNC in MTG

In KEGG enrichment analysis LATE‐NC was characterized primarily by enrichment of lipid and sterol metabolic pathways, inflammatory signaling, and regulatory signaling pathways, especially in astrocytic populations (Figure 4E, Table 3). In contrast, ADNC showed broader enrichment of mitochondrial dysfunction‐related pathways across multiple non‐neuronal cell types, including astrocytes, endothelial cells, microglia, oligodendrocytes, and OPCs.

Overall, KEGG results suggest that LATE‐NC in MTG is associated with astrocyte‐centered lipid metabolism and inflammatory/signaling changes, whereas ADNC is associated with broader mitochondrial and stress‐related pathway involvement across glial and precursor populations (Table 3).

Consistent with the KEGG results, ADNC showed broad enrichment of mitochondrial respiration and oxidative phosphorylation‐related GO terms across astrocytes, microglia, oligodendrocytes, and OPCs (Figure 4F, Table 3).

GO analysis also identified ADNC‐associated enrichment of proteostasis and RNA‐processing terms, including protein folding/refolding, response to misfolded proteins, and RNA splicing, particularly in oligodendrocyte and vascular‐associated populations (Table 3). In contrast, LATE‐NC showed more selective enrichment of regulatory signaling and metabolic processes, including G protein‐coupled receptor (GPCR)‐linked signaling, renin–angiotensin‐related regulation, and lipid/steroid metabolic processes in astrocytic and microglial populations. Additional GO terms further supported cell‐type–specific differences between the two pathological groups, including endosomal transport, sulfur metabolism, immune‐regulatory processes, and cytoplasmic translation (Table 3).

Overall, KEGG and GO analyses showed convergent evidence that ADNC is associated with broad mitochondrial, bioenergetic, proteostatic, and RNA‐processing changes, whereas LATE‐NC is associated with more selective regulatory signaling and lipid/steroid metabolic processes.

To assess the anatomic regional generalizability of the MTG findings, we performed the same GSEA in the A9 region. Overall, A9 recapitulated the major disease‐associated molecular themes observed in MTG, while also exhibiting greater transcriptional heterogeneity and stronger immune‐ and vascular‐associated signatures.

Across comparisons, the LATE‐NC group in A9 showed enrichment of regulatory signaling pathways along with sterol and lipid metabolism and extracellular matrix or vascular‐support processes, consistent with the metabolic and signaling‐associated programs observed in MTG. In contrast, the ADNC group demonstrated prominent enrichment of mitochondrial dysfunction, oxidative phosphorylation alterations, proteostasis stress, and immune activation, which parallels the mitochondrial and protein homeostasis signatures identified in MTG. The LATE‐NC&ADNC group in A9 again did not display a simple intermediate profile but instead showed amplified inflammatory and metabolic stress pathways together with translational dysregulation.

Compared to MTG, A9 exhibited stronger enrichment of immune signaling, vascular remodeling, and stress‐response pathways across disease groups, suggesting region‐specific modulation of shared pathogenic mechanisms.

### 3.5 Cell–cell communication analysis

**FIGURE 5:**
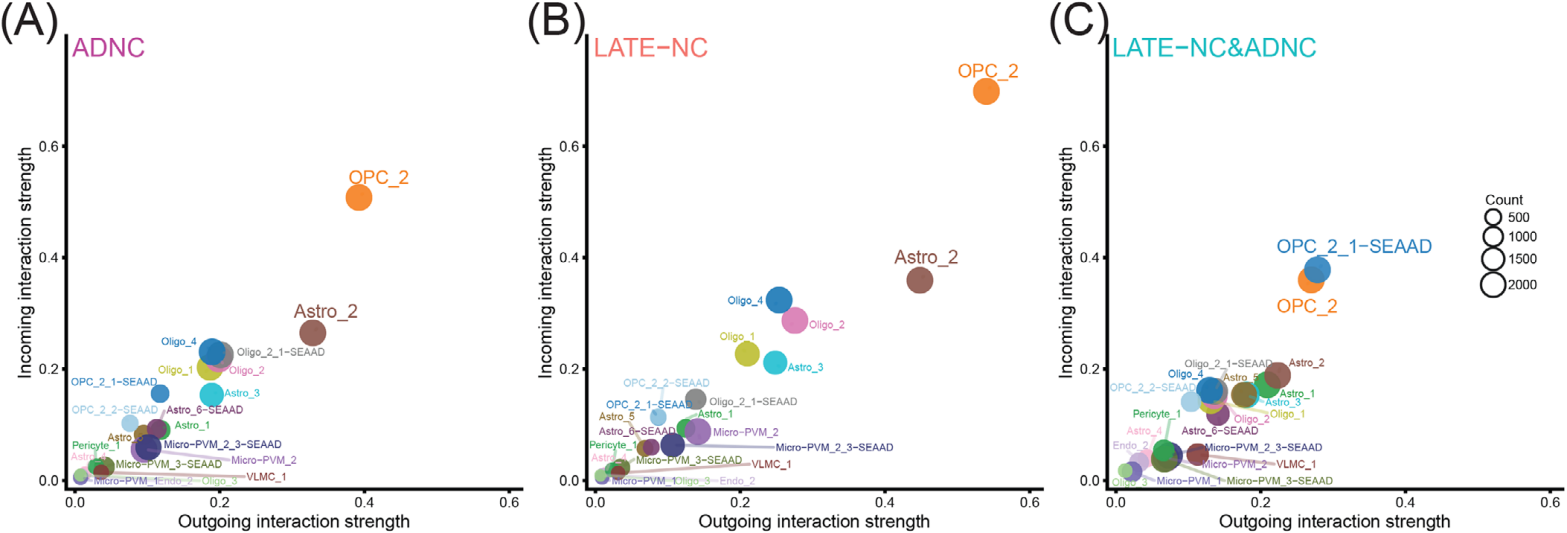
CellChat signaling role analysis of non‐neuronal supertypes in MTG. Signaling role scatter plots showing inferred incoming and outgoing interaction strength for non‐neuronal MTG supertypes in (A) ADNC, (B) LATE‐NC, and (C) LATE‐NC&ADNC groups. Each point represents a cell supertype. The *x* axis indicates outgoing interaction strength, reflecting predicted signal‐sending activity, and the *y* axis indicates incoming interaction strength, reflecting predicted signal‐receiving activity. Point size represents the number of cells in each supertype. ADNC, Alzheimer's disease neuropathologic change; LATE‐NC, limbic‐predominant age‐related transactive response DNA binding protein 43 kDa encephalopathy neuropathological change; MTG, middle temporal gyrus.

To address whether disease‐associated transcriptional changes were accompanied by altered intercellular communication, we performed CellChat analysis across non‐neuronal MTG supertypes. Overall, the total number of inferred interactions was comparable across ADNC, LATE‐NC, and LATE‐NC&ADNC groups, whereas LATE‐NC showed the highest overall inferred interaction strength in the MTG region. We next examined signaling roles to identify major sender and receiver populations. Across pathology groups, OPC and astrocyte supertypes emerged as major communication hubs. In ADNC and LATE‐NC, OPC_2 and Astro_2 showed prominent incoming and outgoing signaling roles, with the strongest hub‐like pattern observed in LATE‐NC. In the LATE‐NC&ADNC, communication shifted toward OPC‐related populations, particularly OPC_2 and OPC_2_1‐SEAAD, while Astro_2 was less dominant than in LATE‐NC (Figure 5 and Figure S14 in supporting information).

Differential sender–receiver analysis further supported pathology‐associated rewiring of non‐neuronal communication. LATE‐NC showed increased OPC_2/Astro_2–centered interactions relative to ADNC, including OPC_2‐to‐OPC_2, Astro_2‐to‐OPC_2, OPC_2‐to‐Astro_2, and Astro_2‐to‐Astro_2 edges (Figure S14). In contrast, LATE‐NC&ADNC showed reduced OPC_2/Astro_2–centered interactions relative to LATE‐NC and increased OPC_2_1‐SEAAD–centered communication, including astrocyte‐to‐OPC_2_1‐SEAAD and OPC‐to‐OPC_2_1‐SEAAD edges (Figure S14). These findings suggest that mixed pathology is associated with reorganization of non‐neuronal communication architecture rather than a simple additive increase in ADNC‐ or LATE‐NC‐associated signaling. A9 shows an overall consistent communication pattern with MTG. In both regions, LATE‐NC shows the strongest overall inferred communication strength, and OPC/astrocyte populations emerge as major signaling hubs (Figure S15 in supporting information).

## 4 DISCUSSION

In this study, we analyzed snRNA‐seq data to compare transcriptional profiles across the LATE‐NC group, the ADNC group, and the LATE‐NC&ADNC group, focusing on non‐neuronal cells. By concentrating on cell type–specific gene expression and pathway‐level perturbations, our analyses highlight both shared and disease‐specific molecular signatures that help clarify the biological heterogeneity underlying clinically overlapping neurodegenerative conditions.

Neuronal subclasses were not included in the differential expression analyses. Instead, the study focused on glial and vascular populations, which have been increasingly recognized as key contributors to neurodegenerative disease biology. Previous studies have identified disease‐associated astrocyte and microglial states in AD^202^, ^203^ and extensive transcriptomic alterations in vascular cells across multiple neurological disorders.^204^, ^205^, ^206^, ^207^, ^208^, ^209^, ^210^ Focusing on these non‐neuronal compartments allowed us to examine disease‐associated transcriptional responses in glial and vascular cell types more directly. The neuronal RNA contamination assessment suggests limited ambient RNA contamination. Slightly higher relative expression was observed in OPC populations, which is consistent with their anatomical proximity to neurons.

Pairwise differential expression analyses identified distinct transcriptional programs associated with each neuropathologic group. The LATE‐NC group was characterized by upregulation of genes involved in cellular signaling (e.g., *PAK1*, ^65^ *NRG1* ^91^), lipid and sterol metabolism (e.g., *LDLR*, ^78^ *SREBF2* ^193^), and regulatory pathways linked to inflammatory and metabolic responses (e.g., *TRL4*, ^98^ *IL6R*, *NDUFA2* ^84^), whereas the ADNC group showed stronger enrichment of genes associated with mitochondrial activity (e.g., *NDUFA2*, ^84^ *MT‐ND2* ^72^), oxidative phosphorylation (e.g., *MT‐ND1* ^72^), and cellular stress responses (e.g., *MT*3^107^). Notably, the LATE‐NC&ADNC group exhibited transcriptional features that were not simply intermediate between the LATE‐NC and the ADNC groups but instead reflected a partially distinct molecular state. This supports the notion that LATE‐NC&ADNC comorbidity may represent a biologically meaningful condition rather than a coincidental overlap of two independent disease processes.

Several representative transcripts illustrate the molecular distinctions between groups. *RPS6* was upregulated in LATE‐NC cases relative to LATE‐NC&ADNC, consistent with its role as a regulator of ribosomal signaling and translational control.^53^ In contrast, mitochondrial Complex I genes (*MT‐ND1, MT‐ND2, MT‐ND4*) were consistently elevated in ADNC cases, reflecting the strong mitochondrial and bioenergetic signatures observed in pathway analyses. The LATE‐NC&ADNC cases also exhibited distinctive transcriptional features that reflect convergent cellular stress pathways. For example, *SQSTM1*,^57^ a central adaptor in autophagy and ubiquitin‐mediated protein degradation, was elevated in mixed cases, consistent with increased proteostatic stress and impaired clearance of misfolded proteins in neurodegenerative disease. Additional transcripts, such as *BAIAP2*, which participates in actin cytoskeleton remodeling and cellular structural regulation,^55^ were also enriched in mixed pathology. This suggests altered cellular remodeling responses under combined disease conditions.

Among non‐neuronal cells, astrocytes and oligodendrocytes emerged as the most transcriptionally responsive populations across comparisons. Astrocytic subtypes showed particularly strong differential expression signals and enrichment of pathways related to signaling regulation, lipid metabolism, and cellular stress responses, which is consistent with their role in maintaining metabolic and homeostatic support functions in the central nervous system. ^211^, ^212^ Oligodendrocytes also showed prominent transcriptional alterations, especially in pathways involving mitochondrial metabolism, protein folding, and proteostasis, highlighting their susceptibility to metabolic and protein QC stress across both pathologies. Overall, these findings suggest that group‐specific, cell type–resolved transcriptional signatures provide a foundation for prioritizing disease‐associated genes that may help inform future efforts to refine diagnostic classification beyond conventional clinicopathologic frameworks. Age and sex are important drivers of neurodegenerative disease risk and molecular pathology.^213^, ^214^, ^215^, ^216^ We conducted preliminary sensitivity analyses by adding interaction terms for age and sex, respectively, in each of the NEBULA models. This change in modeling affected the number of significant DEGs for some supertypes, and these changes were not uniform across cell types or group comparisons. These preliminary findings are summarized in Table S14 in supporting information. We plan to investigate these interactions more comprehensively in future work.

Pathway‐level enrichment analyses further reinforced this distinction. In the MTG, LATE‐NC was preferentially associated with signaling and regulatory pathways, including growth‐factor receptor signaling, GPCR‐linked pathways, and lipid and sterol metabolic processes, which are consistent with altered cellular communication and metabolic regulation in TDP‐43–associated neurodegeneration. ^217^, ^218^ In contrast, ADNC demonstrated dominant enrichment of mitochondrial dysfunction, oxidative phosphorylation, and proteostasis‐related pathways, reflecting well‐established mechanisms of AD progression. ^219^, ^220^ The LATE‐NC&ADNC group exhibited a hybrid but non‐additive profile, marked by dysregulation of ribosomal function, lipid metabolism, and stress‐response pathways. These results indicate that comorbidity introduces additional layers of cellular stress and homeostatic disruption that are not fully captured by either disease alone.

The CellChat analysis provides a tissue‐level extension of the DEG and GSEA results. Rather than showing only that individual glial cell types alter gene expression in association with pathological states, these results suggest that pathology‐associated transcriptional changes are accompanied by altered communication among non‐neuronal cell populations. In the MTG region, ADNC and LATE‐NC shared an overall similar hub structure, with OPC_2 and Astro_2 showing prominent incoming and outgoing signaling roles in both groups. However, LATE‐NC showed stronger overall inferred interaction strength and prominent OPC_2/Astro_2–centered communication, consistent with the signaling and regulatory programs observed in the DEG/GSEA analyses. In contrast, mixed LATE‐NC&ADNC showed a shift toward OPC_2_1‐SEAAD–centered communication, supporting the interpretation that mixed pathology represents a distinct non‐neuronal tissue state rather than an intermediate between ADNC and LATE‐NC.

Although our analyses included two neocortical brain regions, the MTG emerged as the most informative region for distinguishing transcriptional differences across disease groups. MTG showed robust cell type–specific differences in both gene expression and pathway enrichment, particularly within astrocytic, oligodendrocytic, and microglial populations. These findings are consistent with prior reports identifying MTG as a region vulnerable to glial‐mediated transcriptional and metabolic alterations in neurodegenerative disease.^30^, ^221^ While our primary analyses focused on the MTG, complementary analyses in the A9 region (supporting information) revealed stronger immune, vascular, and signaling‐related transcriptional programs across disease groups, with LATE‐NC characterized by enrichment of signaling and lipid/sterol metabolic pathways, ADNC by mitochondrial dysfunction and proteostatic stress, and LATE‐NC&ADNC by amplified inflammatory and metabolic dysregulation.

Several limitations should be considered when interpreting these findings. First, group sizes were not fully balanced across neuropathologic categories, reflecting the inherent challenges of assembling well‐characterized cohorts based on neuropathologic operationalization. Because the LATE‐NC&ADNC group contained more donors than the LATE‐NC group, statistical power may differ across pairwise comparisons, and the number of detected DEGs may partially reflect differences in sample size rather than purely biological effects. Although NEBULA's mixed‐effects framework models donor‐level random effects together with cell‐level variability, which helps mitigate potential overfitting in unbalanced datasets, DEG counts across groups should still be interpreted cautiously. In addition, LATE‐NC stage 3 cases occurred only within the LATE‐NC&ADNC group in this cohort, which prevents the complete separation of LATE‐NC stage effects from concomitant ADNC burden. As a result, some transcriptional differences between LATE‐NC and LATE‐NC&ADNC groups may partially reflect more advanced LATE‐NC staging rather than solely the presence of ADNC. Second, assessment of neuronal RNA contamination indicated that neuronal marker expression in non‐neuronal populations was minimal and consistent with the low ambient RNA background typical of snRNA‐seq datasets. Because even low‐level neuronal transcript carryover may influence pathway‐level enrichment analyses based on ranked gene lists, interpretations of synaptic‐related categories should be made with caution. Third, TDP‐43–associated pathways were observed across multiple disease comparisons rather than being restricted to LATE‐NC cases. Additional analyses comparing minimal‐to‐no ADNC/LATE‐NC samples to each neuropathological group indicated that direct TDP‐43–related KEGG terms were not prominently enriched in the minimal‐to‐no ADNC/LATE‐NC versus LATE‐NC comparisons, while we observed a related proteostasis pathway of valosin‐containing protein–associated 26S proteasome–mediated degradation enriched in the LATE‐NC group compared to the minimal‐to‐no ADNC/LATE‐NC group.^130^ Both ADNC and LATE‐NC&ADNC cases demonstrated enrichment of TDP‐43– and Aβ‐related pathways relative to the minimal‐to‐no ADNC/LATE‐NC group. These findings suggest that TDP‐43–linked transcriptional signatures may not be exclusive to LATE‐NC–dominant pathology and may instead reflect broader interactions among TDP‐43, proteostasis stress, and co‐occurring neurodegenerative processes. Additionally, neither the MTG nor A9 represents the canonical limbic regions most severely affected by TDP‐43 pathology. Therefore, regional sampling may limit sensitivity to mechanisms that are more prominent in the amygdala or hippocampal regions in advanced LATE‐NC. However, we also note that cell‐type substitution in LATE‐NC (due to hippocampal sclerosis) could render problematic an assessment of gene expression changes in the medial temporal lobe regions. Finally, although this study used experimentally generated SEA‐AD single‐nucleus data and retained the SEA‐AD reference annotations, our cell type–specific differential expression findings were derived computationally and were not independently validated with new tissue‐based experiments. Future work will be important to validate prioritized genes in their predicted cell‐type and spatial contexts.

Future studies will benefit from extending this framework to larger and more stage‐balanced cohorts, as well as to brain regions with higher TDP‐43 burden, such as the hippocampus and amygdala. Integration of multi‐omics modalities that include chromatin accessibility and proteomic profiling will be essential for validating the functional consequences of the transcriptional programs identified here. In addition, machine‐learning approaches trained on cell type–specific expression signatures may provide a promising avenue for developing classifiers that distinguish LATE‐NC, ADNC, and mixed pathology in clinical and translational settings.

In summary, this study provides a non‐neuronal cell type–resolved transcriptional framework that highlights both shared and disease‐specific biology for the LATE‐NC group, the ADNC group, and LATE‐NC&ADNC comorbidity. By emphasizing MTG‐specific molecular signatures and contextualizing regional variability, our findings advance understanding of the biological heterogeneity underlying overlapping neurodegenerative pathologies and lay the groundwork for more precise diagnostic and therapeutic strategies.

## CONFLICT OF INTEREST STATEMENT

The authors declare no conflicts of interest. Author disclosures are available in the Supporting Information.

## CONSENT STATEMENT

This study used de‐identified human data from the SEA‐AD consortium. Access to the data was granted upon application and approval. No new human subjects were recruited. Consent was not necessary.

## Supporting information



Supporting Information

Supporting Information

Supporting Information

Supporting Information

Supporting Information

Supporting Information

Supporting Information

Supporting Information

Supporting Information

Supporting Information

Supporting Information

Supporting Information

Supporting Information

Supporting Information

Supporting Information

Supporting Information
