# Supplementary material for: Distinct single‐nucleus RNA‐seq changes among non‐neuronal cells in ADNC, LATE‐NC, and mixed pathologies
Source: Alzheimers Dement. 2026 Sep 23;22(9):e71810. doi: 10.1002/alz.71810 (PMC13601766; doi:10.1002/alz.71810)
Supplement: Supplementary file 2 [file ALZ-22-e71810-s004.docx]

# Supplementary Information: Distinct single-nucleus RNA-Seq changes among non-neuronal cells in ADNC, LATE-NC, and mixed pathologies

Qi Qiao^1,2^, Yuriko Katsumata^2,4^, Hsin-Yu Lai^7^, Kevin Z. Lin^9^, Steven A. Claas^1^, Mariano I. Gabitto^7,8^, Josh M. Morganti^2,5^, Peter T. Nelson^2,3^, Shubhabrata Mukherjee^6^, David W. Fardo^1,2,4^

^1^Department of Biostatistics, College of Public Health, University of Kentucky, Lexington, KY, USA

^2^Sanders-Brown Center on Aging and Alzheimer’s Disease Research Center, University of Kentucky, Lexington, KY, USA

^3^Department of Pathology and Laboratory Medicine, University of Kentucky College of Medicine, Lexington, KY, USA

^4^Division of Biomedical Informatics, University of Kentucky College of Medicine, Lexington, KY, USA

^5^Department of Neuroscience, University of Kentucky, Lexington, KY, USA

^6^Department of Medicine, University of Washington, Seattle, WA, USA

^7^Allen Institute for Brain Science, Seattle, WA, USA

^8^Department of Statistics, University of Washington, Seattle, WA, USA

^9^Department of Biostatistics, University of Washington, Seattle, WA, USA

## Cell type annotation and data quality assessment

**Figure S1.** Quantitative neuropathology marker burden across pathology groups in the middle temporal gyrus. Box plots show the distribution of percent positive area for (A) 6E10/Aβ, (B) AT8/phospho-tau, and (C) pTDP-43 in the MTG across the four pathology groups: Minimal-to-no ADNC/LATE-NC, ADNC, LATE-NC, and LATE-NC&ADNC. Each point represents one donor. Percent positive area was used as the primary quantitative metric because it is normalized to the analyzed tissue area, allowing for a more comparable assessment of regional pathology burden across donors. The pTDP-43 signal in MTG was sparse, with most donors showing zero or near-zero values, consistent with MTG not being a canonical region used to define the LATE-NC stage. Because the quantitative image analysis was performed in the middle temporal gyrus (MTG), rather than in the canonical regions typically used for neuropathologic LATE-NC staging, the regional pTDP-43 burden in this dataset was very low and may not be a representative marker for the global LATE-NC stage.

## Cell type annotation and data quality assessment

**Figure S2.** Marker-gene expression supporting subclass and supertype annotations in MTG and A9. (A) Violin plots showing expression of representative supertype marker genes across SEA-AD annotated MTG supertypes. Marker genes were selected to evaluate expression patterns within major non-neuronal subclasses and to support the transferred supertype annotations used in downstream analyses. (B) UMAP visualization of A9 nuclei colored by annotated supertypes and disease groups, including LATE-NC, ADNC and LATE-NC&ADNC. (C) Dot plot showing expression of canonical subclass marker genes across major non-neuronal subclasses in A9. Dot size represents the percentage of cells expressing each marker, and color intensity represents the average scaled expression level. (D) Violin plots showing expression of representative supertype marker genes across annotated A9 supertypes. For violin plots, each gene is displayed in an individual panel, and expression distributions are shown across the corresponding annotated supertypes.

**Figure S3.** Cell-supertype composition across neuropathology groups and individual donors. (A) Stacked bar plots showing the relative abundance of annotated MTG supertypes across neuropathology groups and within individual donors. The left panel summarizes the average supertype composition for each disease group, including ADNC, LATE-NC and mixed LATE-NC&ADNC, while the right panel shows donor-level supertype composition within each group. (B) Stacked bar plots showing the relative abundance of annotated A9 supertypes across neuropathology groups and within individual donors. For each panel, colors indicate annotated supertypes and bar height represents the proportion of cells assigned to each supertype. Donor-level plots illustrate inter-individual variation in cell-type composition within each neuropathology group.

## Supplementary Methods: Assessment of neuronal RNA contamination

To evaluate potential neuronal RNA contamination in non-neuronal cell populations, we examined the expression of canonical neuronal marker genes across all cell classes. A panel of well-established neuronal markers representing multiple neuronal compartments and functional programs was used, including synaptic vesicle and neurotransmission genes (*SYT1, SNAP25, SYN1*), neuronal identity and cytoskeletal markers (*RBFOX3, STMN2, NEFL, NEFM, NEFH*), excitatory and inhibitory neuronal markers (*SLC17A7, GAD1, GAD2*), neuronal signaling and activity markers (*NRGN, THY1*), and additional neuronal transcripts (*MEG3, GRIN1, DNM1, SNCG*). Average expression of these markers was quantified across all annotated cell supertypes. To assess the relative magnitude of potential contamination, mean expression levels in non-neuronal cell classes were compared to neuronal populations to generate normalized expression ratios. These analyses were used as a diagnostic to distinguish low-level ambient RNA background typical of single-nucleus RNA sequencing from potential cross-cell contamination.

## Assessment of neuronal RNA contamination for the MTG brain region

**Figure S4**. Assessment of neuronal RNA contamination in non-neuronal cell populations for the MTG region. (A) Dot plot showing the expression of canonical neuronal marker genes across non-neuronal cell supertypes. Dot color represents the average normalized expression of each gene, and dot size indicates the percentage of cells expressing the gene. Overall, neuronal marker expression is minimal across non-neuronal cell populations, consistent with low ambient RNA carryover; (B) Dot plot showing the same neuronal marker panel across neuronal cell supertypes, serving as a reference for expected neuronal expression levels. Strong and widespread expression of neuronal markers across neuronal populations confirms the specificity of the selected marker set; (C) Mean average expression of canonical neuronal markers across major non-neuronal cell classes. Expression levels remain low across all non-neuronal populations, with slightly higher values observed in oligodendrocyte precursor cells (OPCs), which are anatomically proximal to neurons; (D) Relative neuronal marker expression in non-neuronal classes normalized to the mean neuronal reference expression. All non-neuronal classes exhibit substantially lower expression levels (typically <5–10% of neuronal levels), supporting minimal neuronal RNA contamination in the analyzed snRNA-seq dataset.

## Assessment of neuronal RNA contamination for the A9 brain region

**Figure S5**. Assessment of neuronal RNA contamination in non-neuronal cell populations for the A9 region. (A) Dot plot showing the expression of canonical neuronal marker genes across non-neuronal cell supertypes. Dot color represents the average normalized expression of each gene, and dot size indicates the percentage of cells expressing the gene. Overall, neuronal marker expression is minimal across non-neuronal cell populations, consistent with low ambient RNA carryover; (B) Dot plot showing the same neuronal marker panel across neuronal cell supertypes, serving as a reference for expected neuronal expression levels. Strong and widespread expression of neuronal markers across neuronal populations confirms the specificity of the selected marker set; (C) Mean average expression of canonical neuronal marker genes across major non-neuronal cell classes in the A9 region. Neuronal marker expression remains low across all non-neuronal populations, indicating minimal neuronal transcript carryover. Slightly higher expression is observed in oligodendrocyte precursor cells (OPCs), consistent with their anatomical proximity to neurons and known low-level background signal in snRNA-seq datasets; (D) Relative neuronal marker expression in non-neuronal classes normalized to the mean neuronal reference expression. All non-neuronal classes exhibit substantially lower expression levels (generally <7% of neuronal expression), supporting that the detected neuronal marker signals represent low-level ambient RNA background rather than substantial neuronal contamination.

## The DEG analysis:

### LATE-NC vs. LATE-NC&ADNC for the A9 brain region

**Figure S6**. (A-B) Volcano plot of DEGs in MTG; (A) genes upregulated in LATE-NC are shown in red and those upregulated in LATE-NC&ADNC in blue. Non-significant genes are indicated in gray; (B) genes upregulated in LATE-NC&ADNC are shown in red, and those upregulated in ADNC are shown in blue. Non-significant genes are indicated in gray. Dashed lines indicate significance thresholds: horizontal line represents an adjusted p-value of 0.05, and the vertical lines represent a fold change of 1.5. Color scheme of the annotated genes: purple: AD-related genes, green: dementia related genes, orange: both AD and dementia related genes based on the Alliance of Genome Resources^1^.

## The DEG analysis:

### LATE-NC vs. LATE-NC&ADNC for the A9 brain region

In A9, differential expression analysis revealed patterns broadly consistent with those observed in MTG with quantitative differences across cell supertypes. Across multiple cell populations, LATE-NC cases exhibited a higher number of DEGs relative to LATE-NC&ADNC, particularly within astrocytic subtypes (e.g., Astro_3). Representative genes upregulated in LATE-NC Astro_3 included *ACHE, AGER, MOBP, RELN, and TAP2*.

**Figure S7**. LATE-NC vs. LATE-NC&ADNC in the A9 region. (A) Number of cells per cell supertype in A9 stratified by diagnosis group. (B) Number of DEGs identified per cell supertype between LATE-NC and LATE-NC&ADNC cases. (C) Volcano plot of DEGs in A9; genes upregulated in LATE-NC are shown in red and those upregulated in LATE-NC&ADNC in blue. Non-significant genes are indicated in gray. Dashed lines indicate significance thresholds: horizontal line represents an adjusted *p*-value of 0.05, and vertical lines represent a fold change of 1.5. Color scheme of the annotated genes: purple: AD-related genes, green: dementia related genes, orange: both AD and dementia related genes based on the Alliance of Genome Resources^1^.

### ADNC vs. LATE-NC&ADNC for the A9 brain region

In A9, differential expression analysis identified relatively limited transcriptional differences between ADNC and LATE-NC&ADNC cases. The Astro_6-SEAAD supertype exhibited the highest number of DEGs, whereas most other glial and vascular populations showed comparatively few changes. *FGF1* was among the genes upregulated in astrocytic subtypes in the LATE-NC&ADNC group.

**Figure S8**. ADNC vs. mixed LATE-NC&ADNC in the A9 region. (A) Number of cells per cell supertype in A9 stratified by diagnosis group (ADNC vs. LATE-NC&ADNC). (B) Number of DEGs identified per cell supertype between ADNC and LATE-NC&ADNC cases in A9. (C) Volcano plot of DEGs; genes upregulated in LATE-NC&ADNC are shown in red and those upregulated in ADNC in blue. Non-significant genes are indicated in gray. Dashed lines indicate significance thresholds: horizontal line represents an adjusted *p*-value of 0.05, and vertical lines represent a fold change of 1.5. Color scheme of the annotated genes: purple: AD-related genes, green: dementia related genes, orange: both AD and dementia related genes based on the Alliance of Genome Resources^1^.

### LATE-NC vs. ADNC for the A9 brain region

In A9, differential expression analysis identified transcriptional differences across astrocytic, microglial, oligodendrocytic, and OPC populations. Patterns were broadly consistent with those observed in MTG, with LATE-NC–dominant cases showing enrichment of metabolism-associated genes, whereas ADNC-dominant cases exhibited stronger representation of mitochondrial and proteostasis-related signatures.

**Figure S9**. LATE-dominant vs. ADNC-dominant in the A9 region. (A) Number of cells per cell supertype in A9 stratified by diagnosis group (LATE vs. AD). (B) Number of DEGs identified per cell supertype between LATE and AD cases in A9. (C) Volcano plot of DEGs; genes upregulated in LATE are shown in red and those upregulated in ADNC in blue. Non-significant genes are indicated in gray. Dashed lines indicate significance thresholds: horizontal line represents an adjusted *p*-value of 0.05, and vertical lines represent a fold change of 1.5. Color scheme of the annotated genes: purple: AD-related genes, green: dementia related genes, orange: both AD and dementia related genes based on the Alliance of Genome Resources^1^.

**Figure S10**. Bar plots showing absolute normalized enrichment scores (|NES|) for selected pathways in MTG. Color intensity corresponds to statistical significance (–log10 adjusted p-value). Pathways are grouped by neuropathologic group for visualization.

## Enrichment Analysis

### Functional pathway differences between LATE-NC and LATE-NC&ADNC in A9

**Figure S11**. KEGG and GO enrichment analysis in A9 (LATE-NC–dominant vs. mixed LATE-NC&ADNC). (A, B) Heatmaps summarizing significantly enriched KEGG pathways (A) and GO terms (B) across cell supertypes. Colored squares indicate significant enrichment in LATE-NC–dominant (salmon) or LATE-NC&ADNC (teal) groups; gray indicates non-significant enrichment. (C) Bar plots showing absolute normalized enrichment scores (|NES|) for selected pathways. Color intensity corresponds to statistical significance (–log10 adjusted *p*-value). Pathways are grouped by disease condition for visualization.

#### KEGG enrichment

In A9, gene set enrichment analysis identified translation initiation–related pathways enriched in both LATE-NC–dominant and mixed LATE-NC&ADNC groups across astrocytic and oligodendrocytic populations. Compared with LATE-NC–dominant cases, LATE-NC&ADNC exhibited broader enrichment of inflammatory and metabolic signaling pathways, including TNF/NFκB, RAS/ERK, and cholesterol biosynthesis across multiple glial and vascular cell types.

#### GO enrichment

GO enrichment analysis in A9 revealed cell-type–specific differences between groups. In astrocytes, LATE-NC–dominant cases showed enrichment of translation- and ribosome-associated processes, whereas mixed LATE-NC&ADNC demonstrated enrichment of stress-responsive, immune-related, and growth factor–associated pathways. Endothelial and vascular-associated populations in LATE-NC&ADNC exhibited enrichment of protein folding, chaperone activity, and junctional organization processes. Microglial populations showed limited enrichment overall, with selective activation of inflammatory signaling pathways in LATE-NC&ADNC. Oligodendrocyte and OPC populations in LATE-NC–dominant cases were enriched for metabolic maintenance processes, while LATE-NC&ADNC showed enrichment of proteostasis-, RNA-processing-, and stress-related pathways.

### Functional pathway differences between ADNC and LATE-NC&ADNC in A9

**Figure S12**. KEGG and GO enrichment analysis in A9 (ADNC-dominant vs. mixed LATE-NC&ADNC). (A, C) Heatmaps summarizing significantly enriched KEGG pathways (A) and GO terms (C) across cell supertypes. Colored squares indicate significant enrichment in ADNC-dominant (purple) or LATE-NC&ADNC (teal) groups; gray indicates non-significant enrichment. (B, D) Bar plots showing absolute normalized enrichment scores (|NES|) for selected pathways. Color intensity corresponds to statistical significance (–log10 adjusted *p*-value). Pathways are grouped by condition for visualization.

#### KEGG enrichment

In A9, KEGG enrichment analysis identified broader pathway representation in mixed LATE-NC&ADNC compared with ADNC-dominant cases. Translation initiation pathways were enriched across multiple non-neuronal cell populations in LATE-NC&ADNC, including astrocytes, oligodendrocyte precursor cells, pericytes, and VLMCs. Microglial populations (e.g., Micro-PVM_2) additionally showed enrichment of mitochondrial dysfunction–related pathways, including electron transport chain–associated signatures. In contrast, ADNC-dominant microglia demonstrated enrichment of selected immune-related signaling pathways.

#### GO enrichment

GO enrichment analysis in A9 revealed functional differences across astrocytic, microglial, oligodendrocytic, and vascular populations. In LATE-NC&ADNC, astrocytes exhibited enrichment of bioenergetic and stress-response processes, including oxidative phosphorylation and ATP-associated pathways. Microglial populations in LATE-NC&ADNC showed enrichment of antigen presentation, mitochondrial, and cytokine-associated processes, whereas ADNC-dominant microglia were enriched for post-translational modification pathways. Oligodendrocyte and OPC populations displayed differences in transport, cytoskeletal, and metabolic processes between groups. Vascular-associated cells demonstrated divergence in metabolic and signaling-related pathways.

### Functional pathway differences between ADNC and LATE-NC in A9

**Figure S13**. KEGG and GO enrichment analysis in A9 (LATE-NC-dominant vs. AD–dominant). (A, C) Heatmaps summarizing significantly enriched KEGG pathways and GO terms across cell supertypes. Colored squares indicate significant enrichment in LATE-NC–dominant (salmon) or ADNC-dominant (purple) groups; gray indicates non-significant enrichment. (B, D) Bar plots showing absolute normalized enrichment scores (|NES|) for selected pathways. Color intensity corresponds to statistical significance (–log10 adjusted *p*-value). Pathways are grouped by condition for visualization.

#### KEGG enrichment

In A9, KEGG enrichment analysis identified distinct pathway distributions between ADNC-dominant and LATE-NC–dominant groups. ADNC-dominant cases demonstrated enrichment of pathways related to translation regulation, proteostasis, calcium-dependent signaling, and mitochondrial electron transport across multiple glial and vascular populations. In contrast, LATE-NC–dominant cases showed enrichment of lipid metabolic pathways, including mevalonate and cholesterol biosynthesis, as well as retrograde transport and endothelial signaling pathways.

#### GO enrichment

GO enrichment analysis further highlighted functional differences between conditions in A9. ADNC-dominant samples exhibited enrichment of protein folding, chaperone-mediated processes, post-translational modification pathways, and immune-associated programs across glial and vascular cell types. In contrast, LATE-NC–dominant samples were enriched for sterol biosynthetic processes, and extracellular matrix–associated pathways in select astrocytic populations.

### Cell-cell communication analysis

**Figure S14**. CellChat visualization of non-neuronal communication networks in MTG. (A–C) Differential interaction circle plots showing pairwise differences in inferred interaction strength among non-neuronal MTG supertypes for LATE-NC&ADNC relative to LATE-NC, LATE-NC&ADNC relative to ADNC, and LATE-NC relative to ADNC. Edge colors indicate the direction of increased inferred communication in each pairwise comparison. (D–F) Heatmaps showing aggregate interaction strength among sender and receiver supertypes in ADNC, LATE-NC, and LATE-NC&ADNC groups. Rows represent source/sender supertypes and columns represent target/receiver supertypes.

### Cell-cell communication analysis

**Figure S15.** CellChat visualization of non-neuronal communication networks in A9. (A–C) Signaling role scatter plots showing outgoing and incoming interaction strength for non-neuronal A9 supertypes in ADNC, LATE-NC, and LATE-NC&ADNC groups. Each point represents a cell supertype; the x-axis indicates outgoing interaction strength, the y-axis indicates incoming interaction strength, and point size represents the number of cells in each supertype. (D–F) Differential interaction circle plots showing pairwise differences in inferred interaction strength for LATE-NC&ADNC relative to LATE-NC, LATE-NC&ADNC relative to ADNC, and LATE-NC relative to ADNC. Edge colors indicate the direction of increased inferred communication in each comparison. (G–I) Heatmaps showing aggregate interaction strength among sender and receiver supertypes in ADNC, LATE-NC, and LATE-NC&ADNC groups. Rows represent source/sender supertypes and columns represent target/receiver supertypes.

## Reference

1.	Consortium, T.A.o.G.R. Alliance of Genome Resources Portal: unified model organism research platform. *Nucleic Acids Research* **48**, D650-D658 (2019).
